# Supplementary figures and images for: Bacterial Infection Drives the Expression Dynamics of microRNAs and Their isomiRs
Source: PLoS Genet. 2015 Mar 20;11(3):e1005064. doi: 10.1371/journal.pgen.1005064 (PMC4368565; doi:10.1371/journal.pgen.1005064)

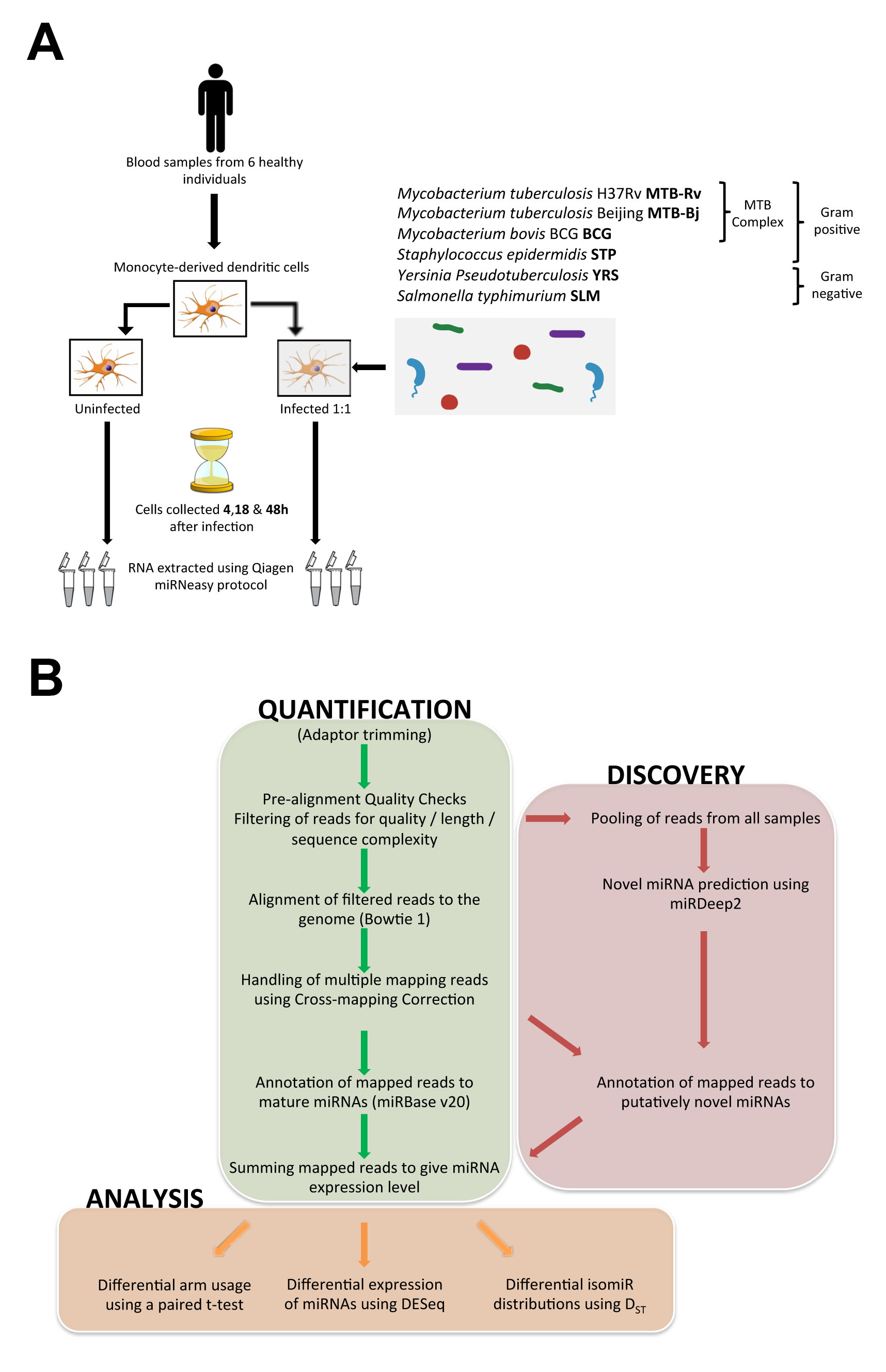

Supplement: S1 Fig — (A) Experimental conditions used and (B) small RNA sequencing data analysis workflow. (TIF) [file pgen.1005064.s001.tif]

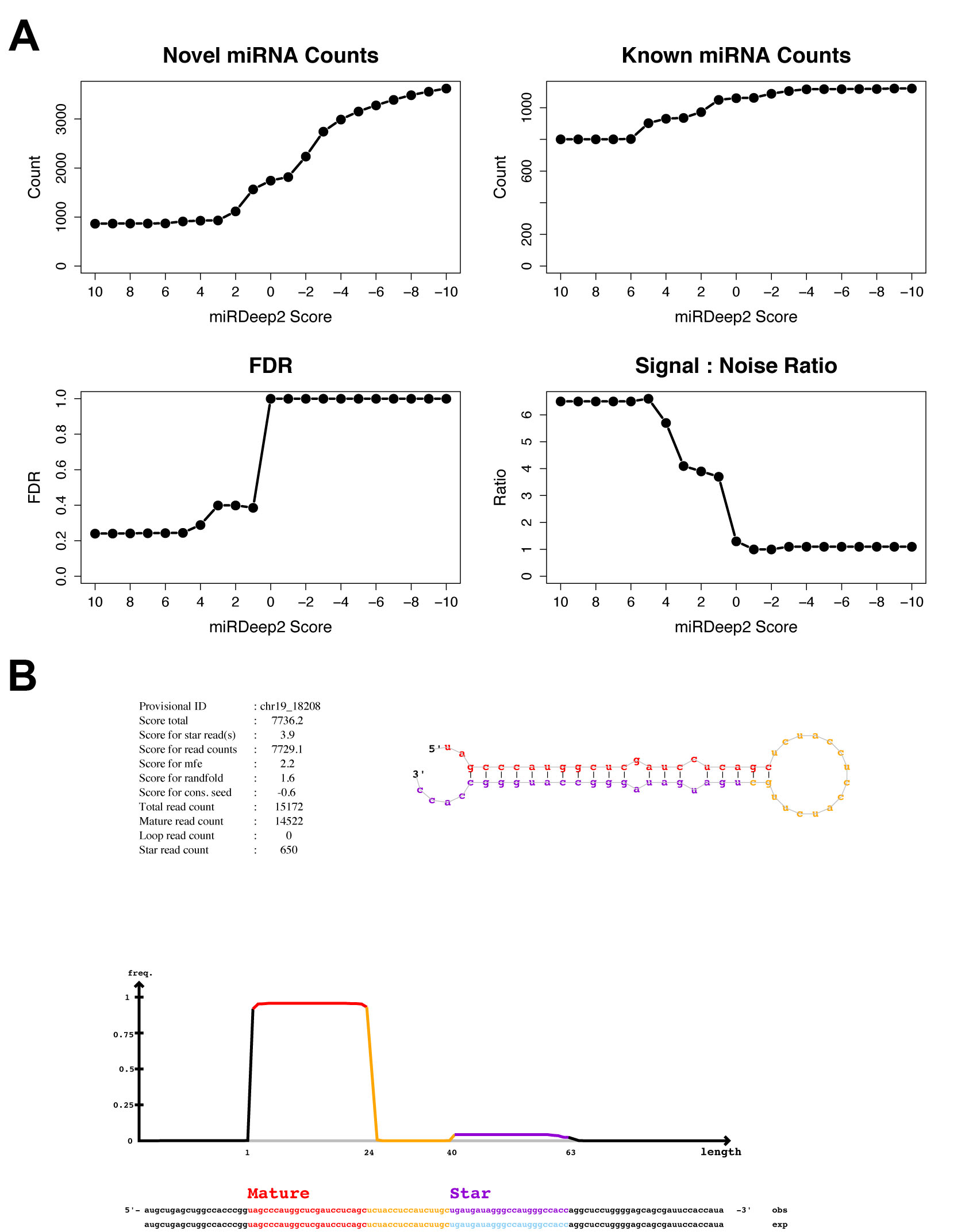

Supplement: S2 Fig — (A) Quality control of miRDeep2 novel miRNA discovery using pooled data from all 116 samples. (B) Example of a high confidence putative novel miRNA identified by our approach. (TIF) [file pgen.1005064.s002.tif]

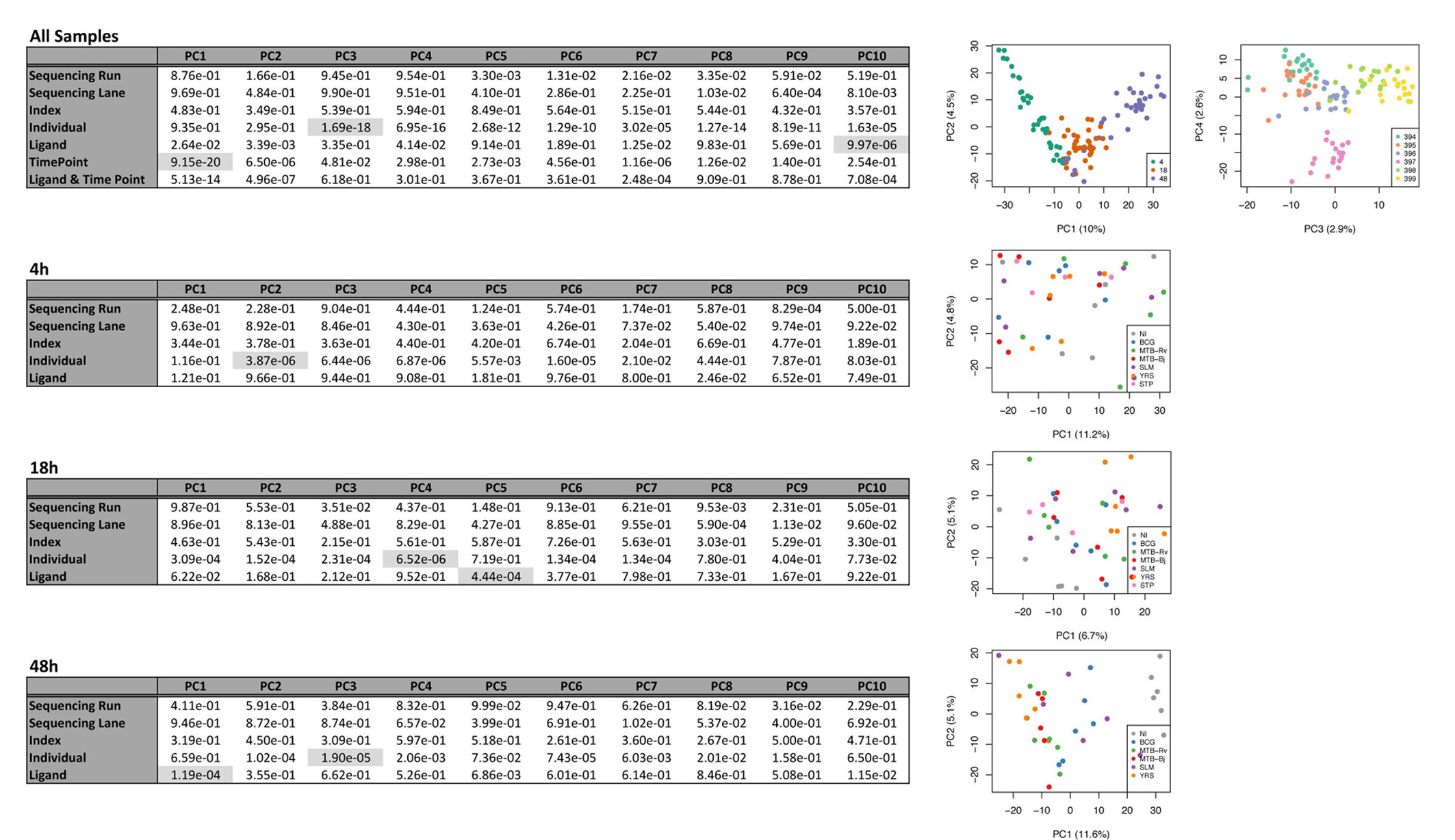

Supplement: S3 Fig — PC analysis was performed on the normalised read counts of 405 robustly expressed miRNAs reported in the results section of this paper. Components were calculated using the R function prcomp, on centred and scaled data. The similarity of the distribution of PCs to known biological variables and potential technical confounders was calculated using a Kruskal-Wallis rank sum test, with the R function kruskal.test. We performed the analysis on all samples together (n = 116) and separately on samples from each of our three time points (4h, 18h and 48h). We found that length of infection accounted for the greatest amount of variance between all samples, consistent with our observation of the increase in the number of differentially expressed miRNAs over time and the results of our clustering analysis (Fig. 1A,B). Consistently, when we repeated PCA on samples from each time point separately we observed that the bacteria with which the samples were infected account for an increasing proportion of the variance with time. Specifically, we observe no correlation of infection with any of the 10 first PCs at 4h, a significant correlation with PC5 at 18h and a significant correlation with PC1 after 48h. We also found, when considering all time points together, a significant correlation between the individual donors and PCs 3 and 4, suggesting a meaningful amount of interindividual variability in miRNA expression. However, we observed no correlation between the PCs and technical variables such as sequencing run, sequencing lane and index sequence, suggesting that these potential technical confounders do not substantially influence our results. (TIF) [file pgen.1005064.s003.tif]

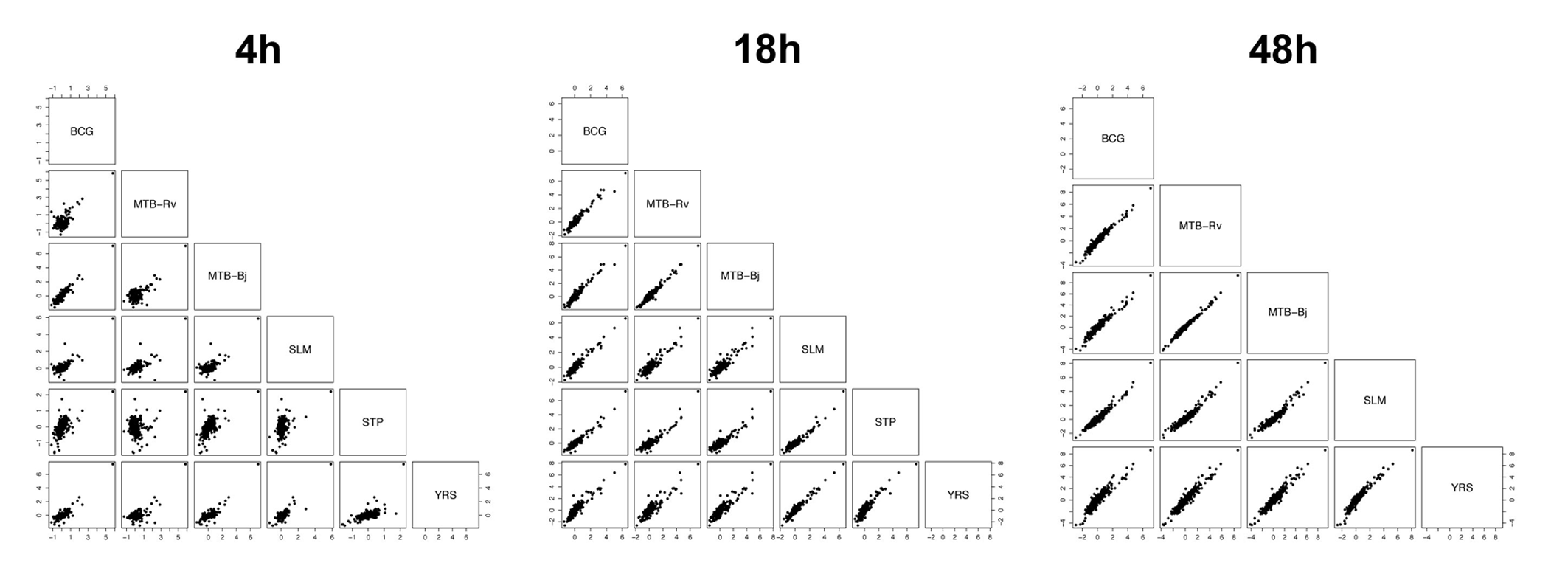

Supplement: S4 Fig — We observed a strong correlation in the fold changes of miRNAs upon infection with our panel of diverse bacteria at 18h and 48h. The correlation was less pronounced at 4h for many comparisons, an observation that may be partially accounted for by the globally smaller fold changes at this early time point. (TIF) [file pgen.1005064.s004.tif]

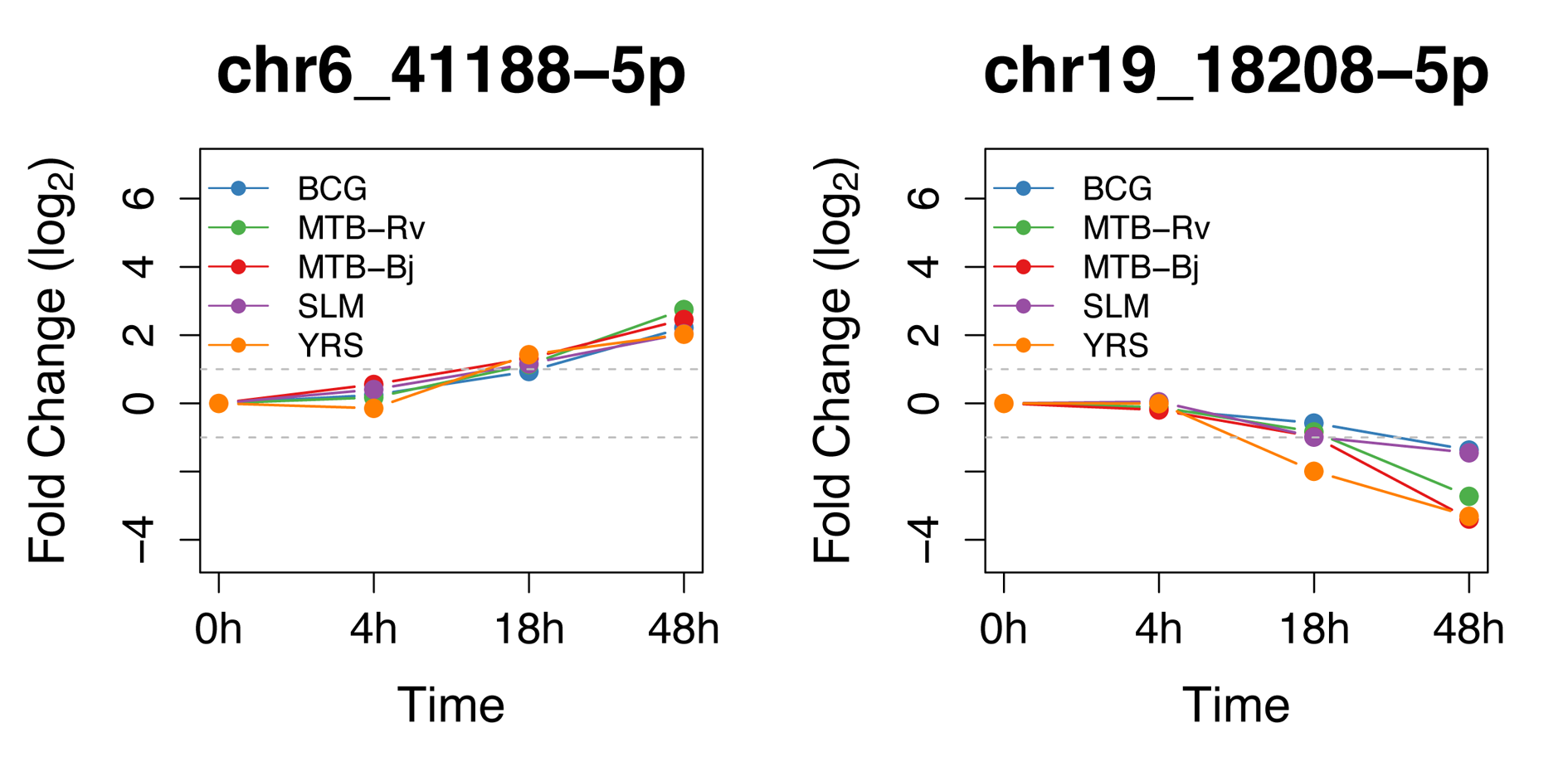

Supplement: S5 Fig — Two putative novel miRNAs (chr6_41188-5p and chr19_18208-5p) that showed highly concordant changes in expression profiles over time across all bacteria. As no expression profiles were available for the 48h time point for STP infection, this bacterium was excluded from the analysis. (TIF) [file pgen.1005064.s005.tif]

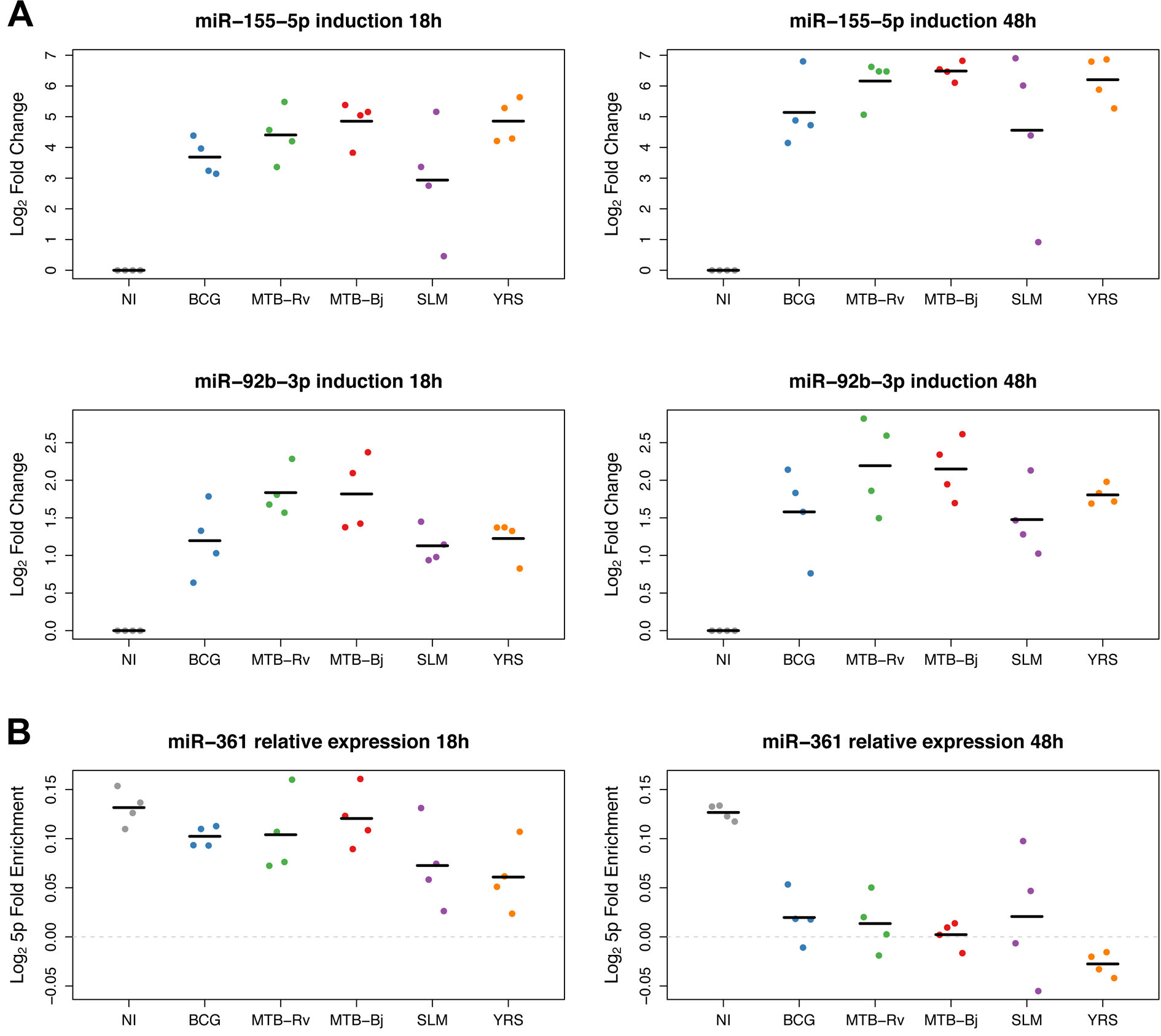

Supplement: S6 Fig — (A) Response to infection of two core miRNAs (miR-155-5p and miR-92b-3p) measured by qPCR. We assessed their induction upon infection with each of our bacteria compared to non-infected cells at the same time point. We confirmed that these miRNAs were significantly induced upon infection with all bacteria at both 18h and 48h. For miR-155-5p, the fold changes were more pronounced at 48h than at 18h, while for miR-92b-3p fold changes were broadly consistent for each bacterium between the two time points, consistent with the temporal induction profiles observed for each by sRNA-seq (Fig. 2A and S3 Table). (B) Relative expression of the arms of the mir-361 hairpin measured by qPCR. We compared their relative expression before and after infection at 18h and 48h. Consistent with our sRNA-seq results, we observed a change in the ratio of the expression of the two arms upon infection. This change was most pronounced at 48h and resulted in a switch in the dominant arm of the miRNA following YRS infection. It should be noted that the fold changes observed by qPCR are much smaller than those obtained by sRNA-seq. This is most likely due to the different way in which expression is measured and calibrated by qPCR, where higher expression is denoted by a lower CT value and normalised to the expression of a housekeeping gene. Importantly, however, the tendencies we observed by sRNA-seq remain the same. The data presented in both panels represent the mean of a duplicate of qPCR calculated for four independent donors (of the six profiled by sRNA-seq). Expression levels were normalised on RNU6-1. The additional two donors as well as the 4h time point were excluded due to limited sample availability. (TIF) [file pgen.1005064.s006.tif]

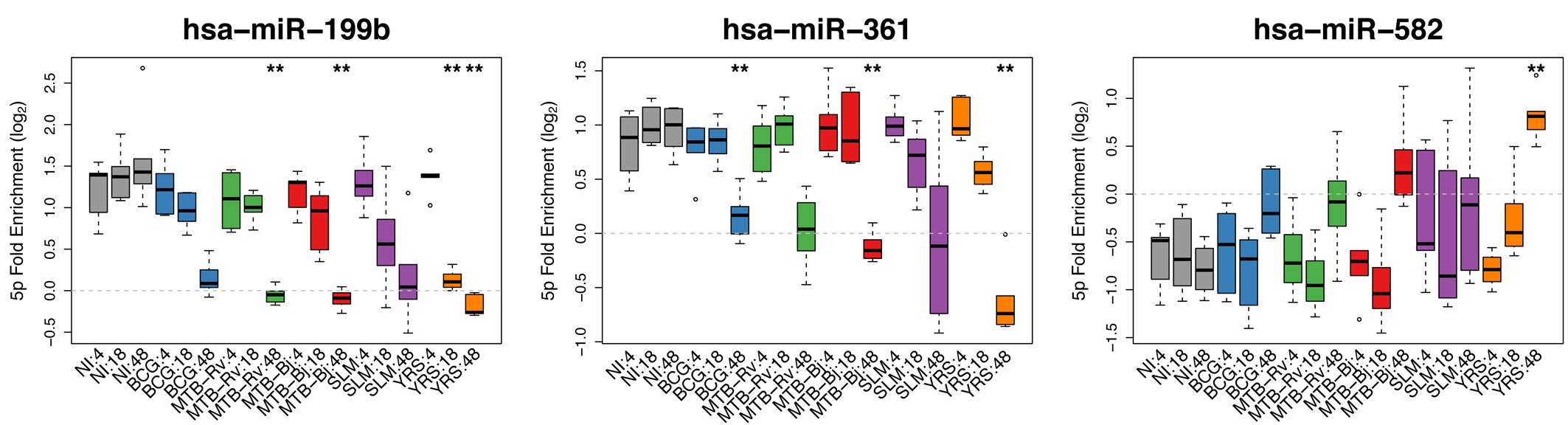

Supplement: S7 Fig — Significance, compared to the non-infected condition, was calculated using a paired t-test (** = FDR-adjusted p < 0.01). (TIF) [file pgen.1005064.s007.tif]

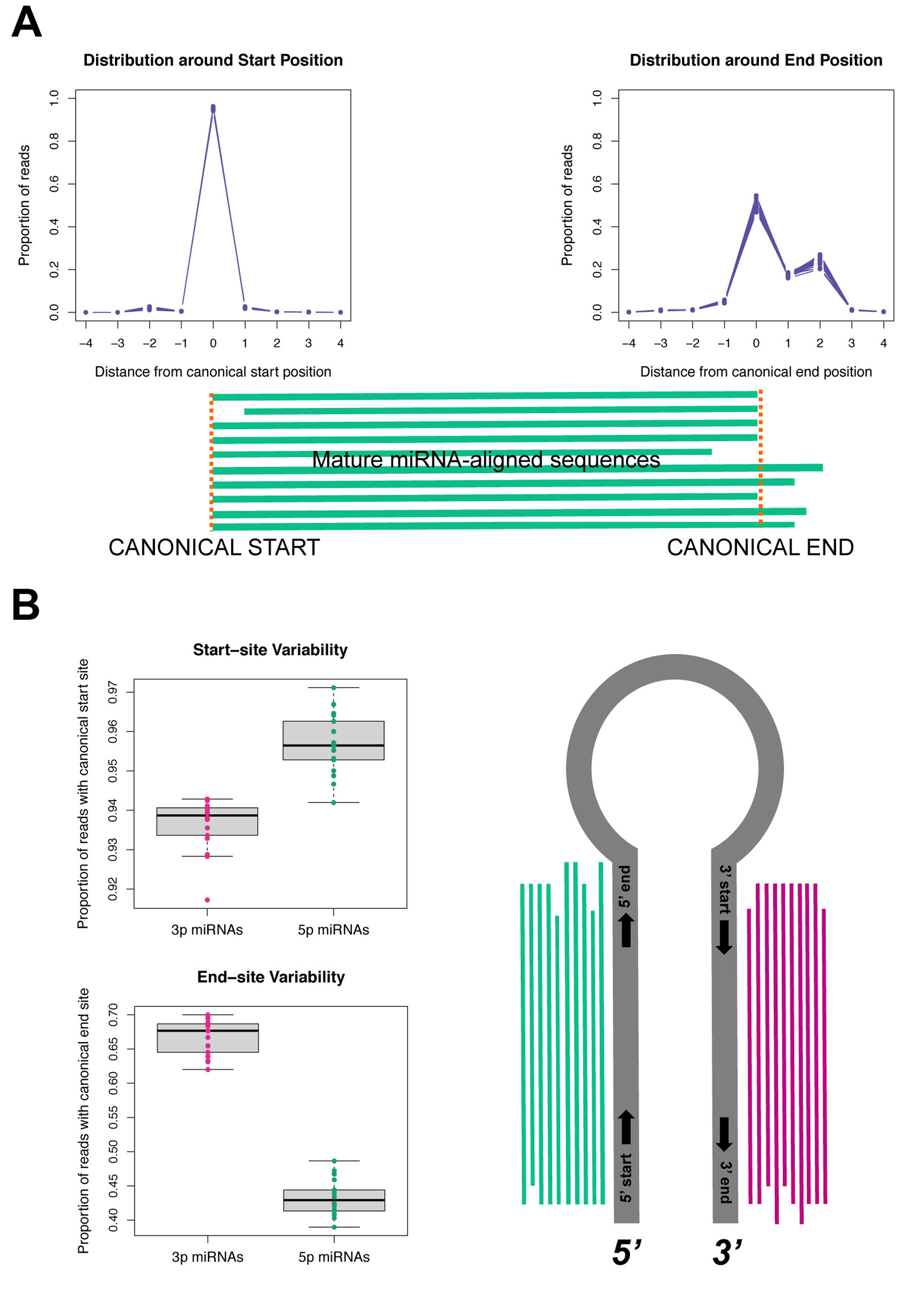

Supplement: S8 Fig — (A) Greater variability was observed at the end of the miRNA sequence, relative to the end-point of the canonical sequence, compared to the start. Specifically, while over 95% of reads aligning to a given miRNA had the same start position as that of the canonical sequence, less than 60% of aligned reads had the same end position as the canonical sequence, with extensions compared to the canonical position more common than shortening. (B) The extent of variability at the start and end sites was differentially distributed between mature miRNAs derived from the 3p and 5p arms of the miRNA hairpin. Specifically, a lower proportion of reads aligning to 3p miRNAs shared the canonical start site compared to reads aligning to 5p miRNAs. Conversely, a lower proportion of reads aligning to 5p miRNAs shared the canonical end site compared to reads aligning to 3p miRNAs. In other words, 3p-derived miRNAs showed relatively greater start-site variability while 5p miRNAs showed relatively greater end-site variability. (TIF) [file pgen.1005064.s008.tif]

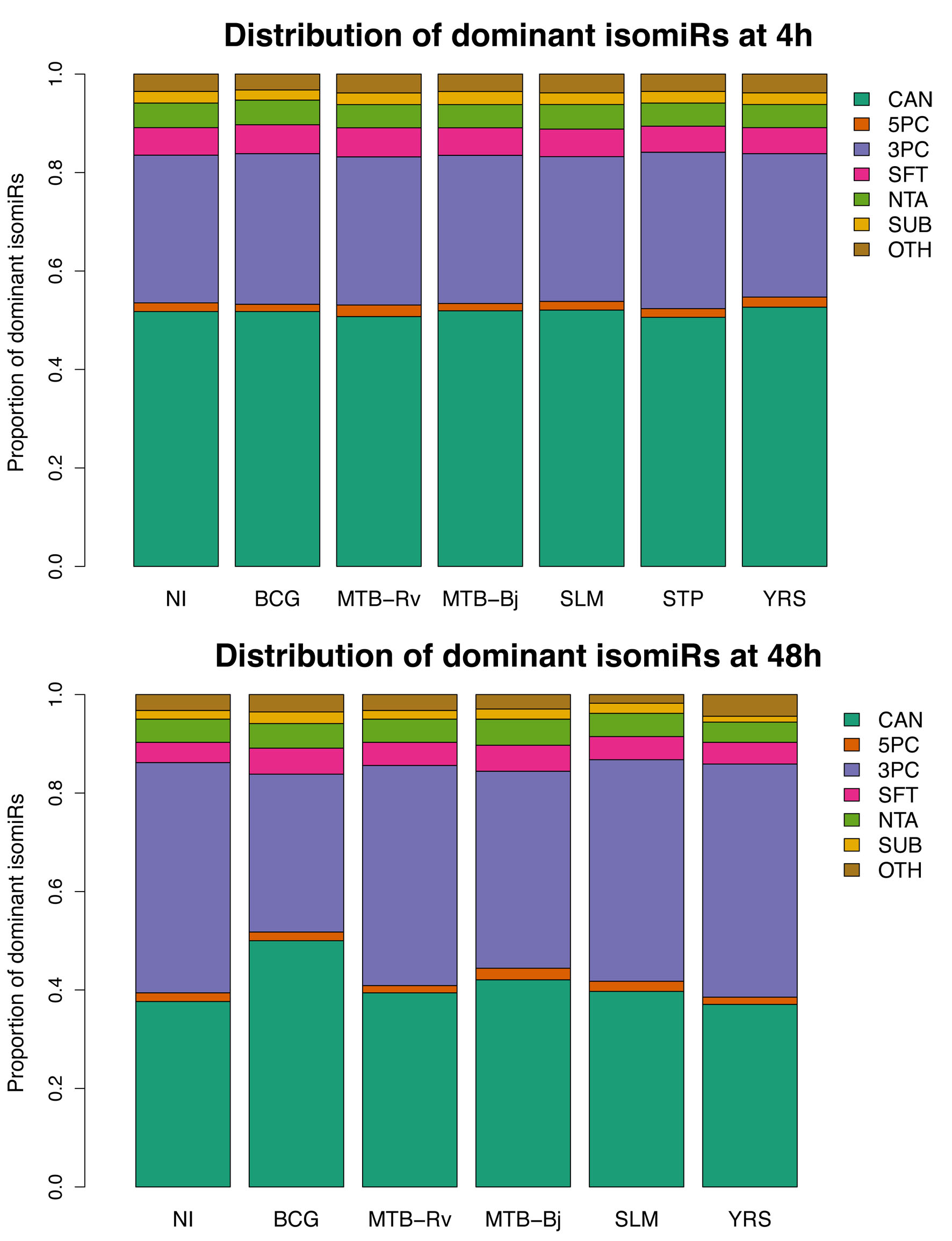

Supplement: S9 Fig — Cumulative barplots showing the proportion of miRNAs for which the dominant sequence was the canonical sequence or, instead, one of six classes of isomiR at 4h and 48h. The results for the 18h time point are presented in Fig. 4B, and a schematic representation of the isomiR acronyms given in the legend is presented in Fig. 4A. (TIF) [file pgen.1005064.s009.tif]

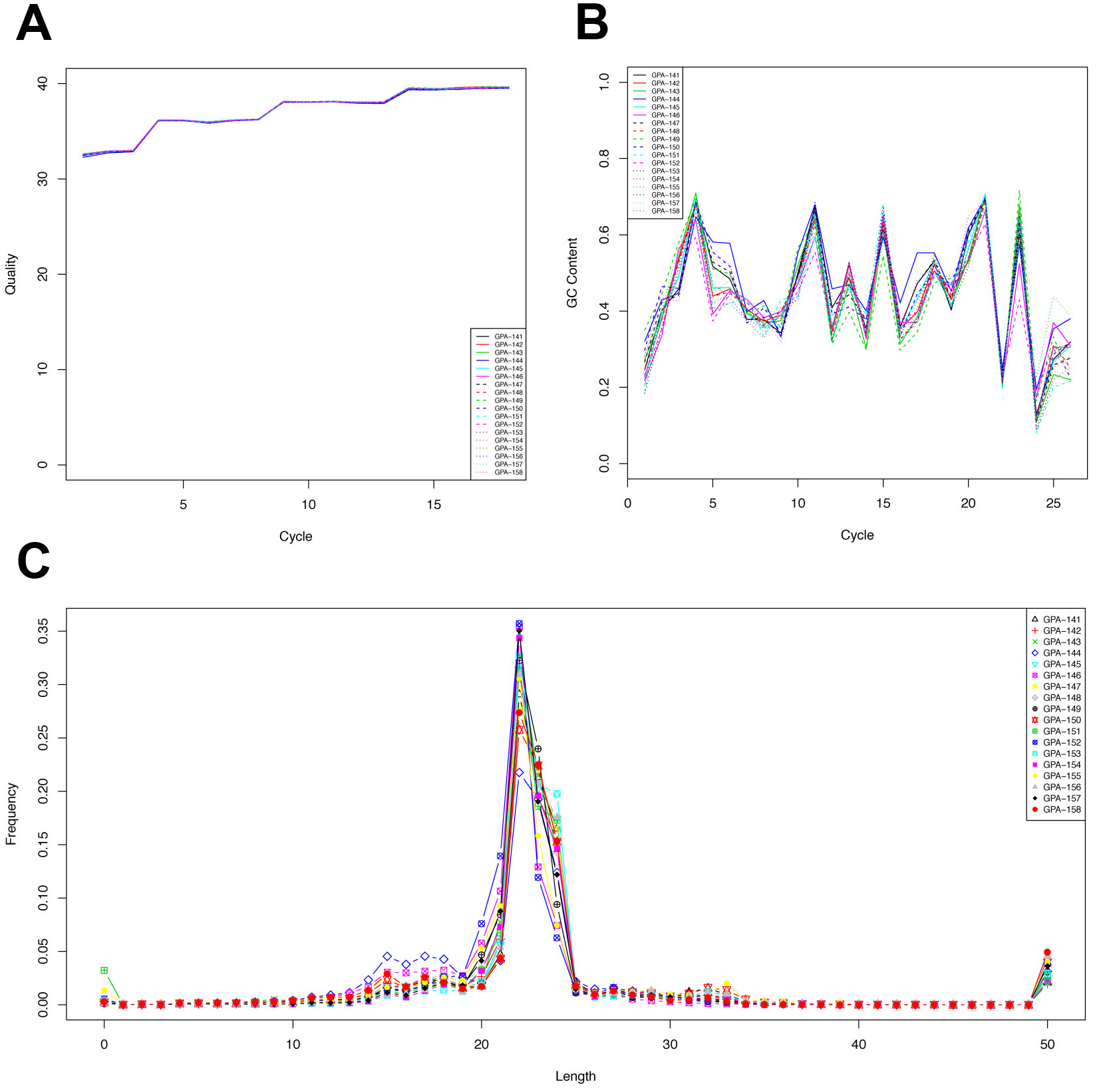

Supplement: S10 Fig — (A) Base quality, (B) GC content distribution and (C) insert lengths of raw sequence reads. Values shown are for one representative sequencing run of 18 samples. All samples were systematically randomised for sequencing to avoid technical confounders that could prevent the detection of true differences between experimental conditions. (TIF) [file pgen.1005064.s010.tif]
