# Supplementary material for: Correction: Bacterial Infection Drives the Expression Dynamics of microRNAs and Their isomiRs
Source: PLoS Genet. 2015 Jun 15;11(6):e1005321. doi: 10.1371/journal.pgen.1005321 (PMC4468252; doi:10.1371/journal.pgen.1005321)
Supplement: S1 File — (PDF) [file pgen.1005321.s001.pdf]

PLOS | GENETICS

RESEARCH ARTICLE

Bacterial Infection Drives the Expression Dynamics of microRNAs and Their isomiRs

Katherine J. Siddle[1,2☯], Ludovic Tailleux[3☯], Matthieu Deschamps[1,2,4], Yong-Hwee Eddie Loh[1,2], Cécile Deluen[5], Brigitte Gicquel[3], Christophe Antoniewski[6], Luis B. Barreiro[7], Laurent Farinelli[5], Lluís Quintana-Murci[1,2]*

1 Institut Pasteur, Unit of Human Evolutionary Genetics, Paris, France, **2** Centre National de la Recherche Scientifique, Paris, France, **3** Institut Pasteur, Unit of Mycobacterial Genetics, Paris, France, **4** Université Pierre et Marie Curie, Cellule Pasteur UPMC, Paris, France, **5** Fasteris SA, Plan-les-Ouates, Switzerland, **6** Université Pierre et Marie Curie, Laboratory of Developmental Biology, Paris, France, **7** Ste-Justine Hospital Research Centre and Department of Paediatrics, Faculty of Medicine, University of Montréal, Montréal, Canada

☯ These authors contributed equally to this work.
* quintana@pasteur.fr

CrossMark
click for updates

OPEN ACCESS

Citation: Siddle KJ, Tailleux L, Deschamps M, Loh Y-HE, Deluen C, Gicquel B, et al. (2015) Bacterial Infection Drives the Expression Dynamics of microRNAs and Their isomiRs. PLoS Genet 11(3): e1005064. doi:10.1371/journal.pgen.1005064

Received: October 8, 2014

Accepted: February 9, 2015

Published: March 20, 2015

Copyright: © 2015 Siddle et al. This is an open access article distributed under the terms of the Creative Commons Attribution License, which permits unrestricted use, distribution, and reproduction in any medium, provided the original author and source are credited.

Data Availability Statement: The miRNA expression data reported in this manuscript have been submitted to the NCBI Gene Expression Omnibus (GEO; http://www.ncbi.nlm.nih.gov/geo/) under the accession number GSE64142.

Funding: The laboratory of LQM has received funding from the Institut Pasteur, the Centre Nationale de la Recherche Scientifique (CNRS), the French Government's Investissement d'Avenir program, Laboratoire d'Excellence "Integrative Biology of Emerging Infectious Diseases" (grant no. ANR-10-LABX-62-IBEID), and from the European Research Council under the European Union's Seventh Framework Programme (FP/2007–2013)/ERC Grant Agreement No. 281297. The laboratory of LT has

Abstract

The optimal coordination of the transcriptional response of host cells to infection is essential for establishing appropriate immunological outcomes. In this context, the role of microRNAs (miRNAs) – important epigenetic regulators of gene expression – in regulating mammalian immune systems is increasingly well recognised. However, the expression dynamics of miRNAs, and that of their isoforms, in response to infection remains largely unexplored. Here, we characterized the genome-wide miRNA transcriptional responses of human dendritic cells, over time, to various mycobacteria differing in their virulence as well as to other bacteria outside the genus *Mycobacterium*, using small RNA-sequencing. We detected the presence of a core temporal response to infection, shared across bacteria, comprising 49 miRNAs, highlighting a set of miRNAs that may play an essential role in the regulation of basic cellular responses to stress. Despite such broadly shared expression dynamics, we identified specific elements of variation in the miRNA response to infection across bacteria, including a virulence-dependent induction of the miR-132/212 family in response to mycobacterial infections. We also found that infection has a strong impact on both the relative abundance of the miRNA hairpin arms and the expression dynamics of miRNA isoforms. That we observed broadly consistent changes in relative arm expression and isomiR distribution across bacteria suggests that this additional, internal layer of variability in miRNA responses represents an additional source of subtle miRNA-mediated regulation upon infection. Collectively, this study increases our understanding of the dynamism and role of miRNAs in response to bacterial infection, revealing novel features of their internal variability and identifying candidate miRNAs that may contribute to differences in the pathogenicity of mycobacterial infections.

received funding from the National Institutes of Health (grant no. NIH AI087658) and the European Commission, within the 7th Framework Programme, (grant no. HEALTH-F3-2009-241745). KJS is a scholar of the Pasteur – Paris University (PPU) International PhD program and was supported by a stipend from the Direction Générale de l'Armement (DGA). YHEL is supported by fellowships from the Fondation pour la Recherche Médicale (FRM) and the Institut Pasteur. The funders had no role in study design, data collection and analysis, decision to publish, or preparation of the manuscript.

Competing Interests: The authors have declared that no competing interests exist.

Author Summary

MicroRNAs (miRNAs) are small, non-coding RNAs that regulate important cellular processes by inhibiting the expression of gene targets. In recent years, it has become clear that miRNAs play a critical role in the regulation of the immune response to infection, a highly complex phenotype involving the activation of both generic and infection-specific responses. However, it remains unclear to what extent miRNAs are involved in the regulation of these two types of response. Here, focusing on the miRNA response to mycobacteria, pathogens of major public health importance, we present the first comparative, deep sequencing-based analysis of the miRNA response to a panel of bacterial infections. We define a set of miRNAs that play an essential role in basic cellular responses to stress and identify pathogen-specific miRNA responses that reflect mechanisms by which certain pathogens interfere with the host response to infection. In addition, we show that infection can alter the expression level and proportions of miRNA isoforms, transcripts originating from the same miRNA but with slight differences in their nucleotide sequences. This study highlights a novel aspect of miRNA expression dynamics upon infection and increases our understanding of miRNA-mediated mechanisms involved in host cellular responses to infection.

Introduction

The response of host cells to microbial infection or immune activation is among the most-well studied examples of cellular responses to external stimuli. This response is characterised by marked changes in gene expression [1–6], which require precise coordination to establish appropriate immunological outcomes, ensuring maximal protection against infection while avoiding tissue damage. The crucial role of microRNAs (miRNAs) – small regulatory RNAs that mediate degradation or translational repression of thousands of target mRNAs [7–9] – in regulating mammalian immune systems is increasingly well established. MiRNAs regulate the development and function of immune cells and can have pro-inflammatory or anti-inflammatory effects [10–14]. Furthermore, experimental data indicate that microbial infection alters the miRNA repertoire of host cells [15,16] and that, when aberrantly expressed, miRNAs can contribute to immunity-related pathological conditions, such as infectious or inflammatory diseases, autoimmunity or cancer [12,14,17,18].

The advent of next-generation sequencing, in particular RNA-sequencing, has enabled the exploration of a myriad of novel questions related to miRNA diversity. For example, besides the detection of many novel miRNAs and the description of an increasingly broad array of non-canonical biogenesis pathways producing functional miRNAs [19–21], RNA-seq studies have highlighted the highly dynamic relative abundance of the 5p and 3p arms of the miRNA duplex, a process known as arm-switching [22]. Indeed, following cleavage by Dicer, the miRNA hairpin produces a ~22-bp RNA duplex, one strand of which is preferentially incorporated into the RNA-induced silencing complex (RISC) as a mature, functional miRNA, whereas the other strand has often been thought to be degraded [23]. Previously believed to be static and dictated by the thermodynamic and structural properties of the duplex [24,25], the choice of the dominant miRNA arm has recently been shown to be flexible across species, tissues and developmental stages [22,26–31].

Deep sequencing has also revealed the presence of sequence variation among mature miRNAs – known as isomiRs – shifting the view of miRNAs from single sequences to heterogeneous repertoires of multiple isoforms [32–34]. For a given miRNA, the distribution of

different isomiRs appears to be non-random and can differ between tissues and developmental stages [27,28,30,35–41]. Moreover, that isomiRs appear to act co-operatively with canonical miRNA sequences, targeting common pathways to reduce the signal-to-noise ratio of mRNA targeting [28], suggests that changes in their proportions may have functional implications for gene regulation. Although these studies indicate that arm dominance and isomiR expression are dynamic, the extent to which they can be altered by external stimuli, such as infection, remains unknown.

The characterisation of the host miRNA responses to bacteria has progressed at a slower pace than that of viral and parasitic infections [16], despite the fact that a number of bacteria are responsible for some of the most devastating infectious diseases today. Notable among these is *Mycobacterium tuberculosis* (MTB), the aetiological agent of tuberculosis (TB), the most deadly disease caused by a single bacterial agent [42]. A large number of miRNAs have been recently described as being involved in the response to MTB and other mycobacteria [43–55]. However, the highly heterogeneous nature of these studies—i.e., the use of different mycobacteria, experimental settings (patients, human cells, mice), cell types or tissues, and times post-infection—has precluded any comparison among them, so a clear understanding of the miRNA transcriptional response to MTB is missing. More generally, the extent to which alterations in miRNA expression upon infection are specific to particular pathogens or strains, or instead reflect general responses of host cells to infection, cell activation or inflammation remains to be explored.

Here, we use deep sequencing to characterise the miRNA transcriptional response over time of a key immune cell type – the dendritic cell (DC) – to various mycobacterial strains differing in their virulence as well as to other intracellular bacteria outside the *Mycobacterium* genus. This global, unbiased approach provides a truly comparative picture of the miRNA repertoire, including novel miRNAs, involved in immunity to MTB and, more broadly, bacteria. We defined core bacterial miRNA responses, as well as responses shared between smaller groups of pathogens or detected in a single condition that may reflect particular mechanisms of virulence or suppression. Furthermore, we explored, for the first time, the extent to which infection impacts both the relative abundance of the arms of the miRNA hairpin and the expression dynamics of miRNA isoforms.

Results

Expression of annotated and novel miRNAs in dendritic cells

To assess the variability of the genome-wide miRNA response to infection, we exposed human monocyte-derived DCs from healthy donors to a diverse set of bacterial species. This panel included three bacteria of the *Mycobacterium tuberculosis* complex (MTBC), a group of closely related mycobacteria that cause TB in humans or other species. Specifically, we used two virulent strains of *Mycobacterium tuberculosis* – the reference strain H37Rv of the Euro-American lineage (MTB-Rv) and a member of the Beijing strain of the East Asian lineage (MTB-Bj) – as well as the attenuated strain *Mycobacterium bovis* BCG (BCG), widely used as a vaccine against TB. In addition, we included a Gram-positive species, *Staphylococcus epidermidis* (STP), and two Gram-negative bacteria, *Salmonella typhimurium* (SLM) and *Yersinia pseudotuberculosis* (YRS) (S1 Fig.). To study variation in miRNA transcript levels at high resolution, we performed small RNA-sequencing (sRNA-seq) from matched non-infected and infected cells at three time points (4h, 18h and 48h). In total, we generated 1.1 billion reads of 50 bp, corresponding to 116 samples with an average of 9.1 million reads per sample after filtering (GSE64142). Of these, 98% were mappable to the human genome while 85% of reads mapped to miRNAs in miRBase v20 (www.mirbase.org).

Following processing of the data (Methods and S1 Fig.), we detected 387 annotated miRNAs that were present in three or more donors in at least one experimental condition (i.e. cells infected with a given bacterium, or left uninfected, at a given time point) at a depth $\geq$ 50 reads (S1 Table). To identify putative novel miRNAs, we applied a two-step discovery and quantification approach using miRDeep2 [56] (see Methods). Of the 369 predicted hairpins, we detected 18 putative mature miRNAs that were present at a depth $\geq$ 50 reads in three or more donors in at least one experimental condition (S2 Fig.). Of these, 5 corresponded to known snoRNAs, 8 were located in introns, 4 were antisense to genes and 1 was intergenic (S2 Table). Together, this dataset presents a comprehensive, unbiased characterisation of the miRome of steady-state and activated DCs.

Highly overlapping miRNA responses to diverse bacterial infections

To identify miRNAs whose expression was altered after bacterial infection, we compared infected samples to the corresponding non-infected time point using the package DESeq [57]. A total of 152 miRNAs (38%), of which 145 were previously annotated and 7 were novel, were significantly differentially expressed (FDR-adjusted $p<0.01$ and $|\log_2 \text{fold change}|>1$) upon encounter with at least one of the six bacteria. For all bacteria, the number of differentially expressed miRNAs increased over time (Fig. 1A and S1 Table). The bacteria that showed the greatest impact on miRNA expression were YRS, MTB-Bj and MTB-Rv ("high-responders"), while BCG, SLM and STP elicited more modest responses ("low-responders") (Fig. 1A). In addition, unsupervised hierarchical clustering clearly separated experimental conditions into three distinct clusters corresponding to the length of infection, with all non-infected conditions clustering with the 4h time point (Fig. 1B), consistent with principle component analysis (S3 Fig.). Overall, these results suggest that the length of infection is a stronger driver of the miRNA response than the identity of the bacterium.

To qualitatively assess the similarity of miRNA responses to the various bacterial infections, we studied the overlap of differentially expressed miRNA sets. We found that over 30% of miRNAs were differentially expressed upon infection with five or more different bacteria, with over 80% shared between at least two independent infections (Fig. 1C). To avoid inflating the dissimilarities between bacteria due to slight differences in fold changes, which were generally highly correlated (S4 Fig.), we also defined miRNAs as significantly differentially expressed when the absolute $\log_2$ fold change was less than 1 if in at least one other experimental condition the change upon infection exceeded this cut-off. Using this threshold, 64% of differentially expressed miRNAs were altered upon infection with at least five of the six bacteria (Fig. 1C). Consistent with their close genetic similarity, MTBC bacteria showed highly overlapping miRNA responses (Fig. 1D). At the same time, less than 10% of miRNAs differentially expressed following MTB-Rv infection were unique to this bacterium, taken as a representative of the MTBC, when compared to more distantly related, non-mycobacterial infections (Fig. 1E). Overall, these results suggest a remarkably consistent miRNA response across diverse bacterial pathogens.

Temporal miRNA dynamics identifies a core response to bacterial infection

We next investigated whether miRNAs that were differentially expressed upon infection showed similar temporal responses across bacteria, using the Short Time-series Expression Miner (STEM) [58,59]. This program, specifically designed for short time-series datasets, uses the changes in expression observed at each time point to cluster miRNAs according to a set of pre-determined model temporal response profiles. We identified 14 miRNAs that were

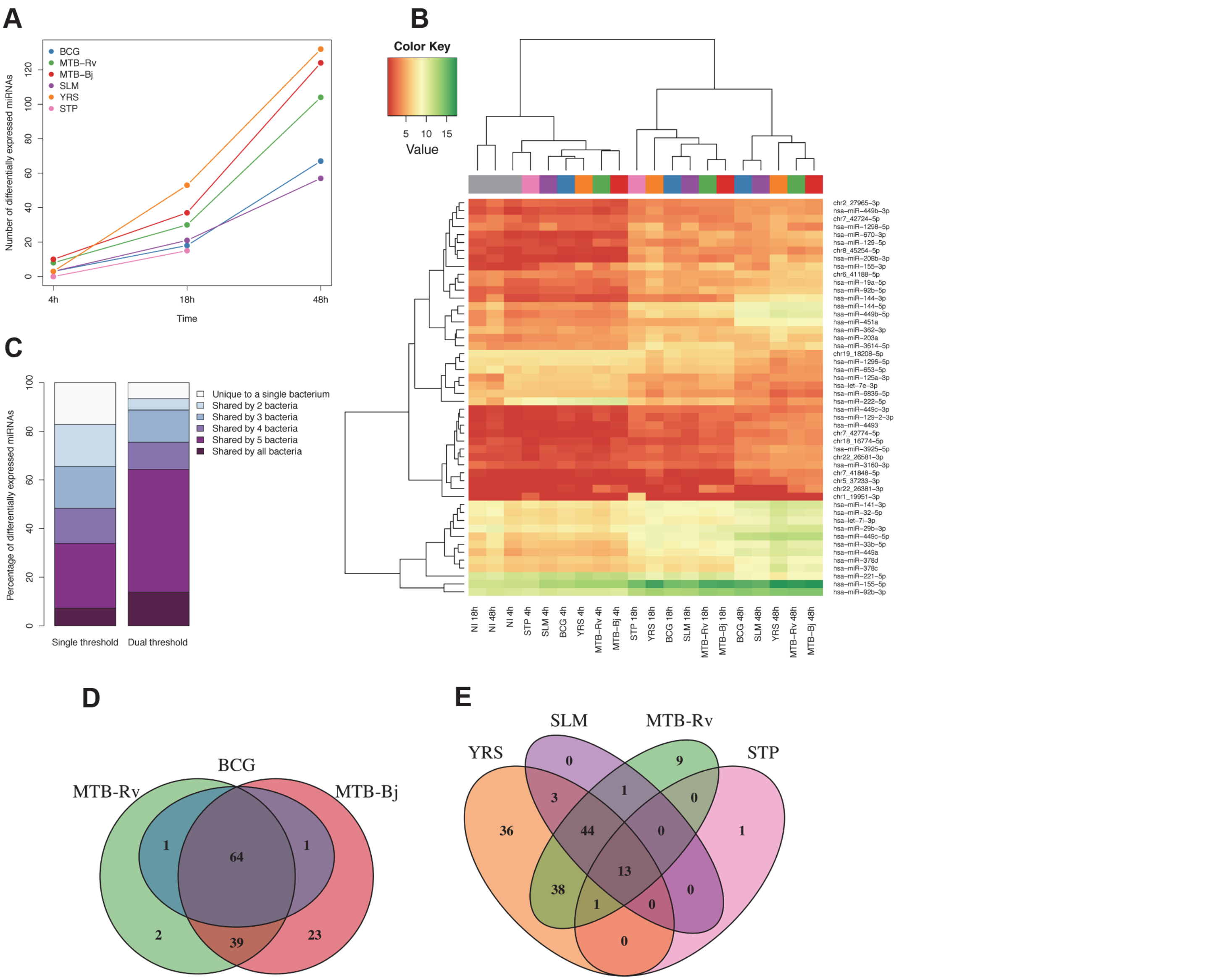

Fig 1. Differential expression of miRNAs in DCs upon infection with a panel of bacteria. (A) Numbers of significantly differentially expressed miRNAs upon infection at each time point for each bacterium. As we did not have expression profiles for the 48h time point for STP infection, this point is missing from the plot. (B) Heatmap illustrating the hierarchical clustering of experimental conditions based on the mean expression levels of the 50 most variable miRNAs. (C) Overlap of differentially expressed miRNAs between bacteria using two different significance cut-offs. Left-bar shows overlap using a single cut-off of FDR-adjusted $p<0.01$ and $|\log_2$ fold change$|>1$, while the right bar shows the overlap using a secondary cut-off where a miRNA was called as significant if the absolute $\log_2$ fold change was less than 1, if it passed the first more stringent fold-change cut-off upon infection with at least one of the six bacteria. (D and E) Venn diagrams showing the overlap of significantly differentially expressed miRNAs between bacteria of the MTBC (D) and between MTB-Rv and all other non-mycobacterial infections (E).

doi:10.1371/journal.pgen.1005064.g001

assigned to the same model profiles in all bacteria and 35 additional miRNAs that were assigned to highly correlated model profiles in all bacteria (see Methods, Fig. 2A and S3 Table). These 49 miRNAs, which represent the basis of the core miRNA response to bacterial infection in DCs, comprised 27 miRNAs that were upregulated upon infection, 21 that were downregulated and one, miR-222-5p, which was upregulated at 4h but downregulated at later time points. In addition, two novel miRNAs (chr6_41188-5p and chr19_18208-5p) were assigned to highly correlated model profiles in all bacteria (S5 Fig.). To check that these core response

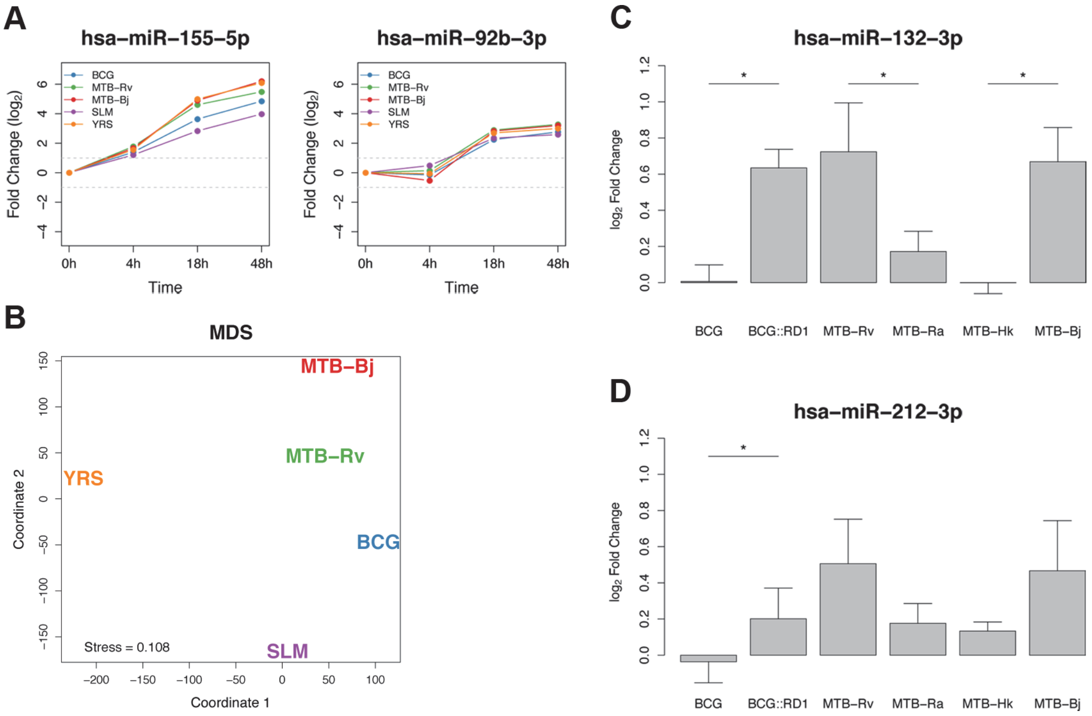

Fig 2. Shared and specific miRNA responses to bacterial infection. (A) Plots of the temporal dynamics of two core response miRNAs. As we did not have expression profiles for the 48h time point for STP infection, this bacterium was excluded from the analysis. (B) Multidimensional scaling analysis (MDS) representing the distances between the temporal miRNA responses to different bacterial infections. Distances were based on the sum of edit distances, for each miRNA, between bacteria using STEM-assigned model temporal profiles. (C) The expression of miR-132-3p increased following infection with MTB-Rv and MTB-Bj but not BCG. Transformation of BCG with RD1 from MTB resulted in a significant increase in miR-132-3p expression, not significantly different to that induced by MTB-Rv. Both MTB-Ra and MTB-Hk showed significantly lower miR-132-3p expression than virulent mycobacteria. (D) The induction of miR-212-3p was significantly higher following BCG::RD1 infection, compared to infection with control BCG, and was not significantly different to the response to MTB-Rv. Though the difference between the induction of this miRNA upon infection with virulent or avirulent MTB strains was not significant, the tendencies observed were consistent with miR-132-3p. Significance was calculated using a Mann-Whitney test (* = $p < 0.05$).

doi:10.1371/journal.pgen.1005064.g002

miRNAs indeed showed consistent responses across bacteria, we chose to test two of them – miR-155-5p and miR-92b-3p – by qPCR and confirmed our observations (S6A Fig.).

To provide insight into the impact of this core miRNA response on innate immune cell function, we performed gene ontology category enrichment analysis for gene targets of core miRNAs as predicted by TargetScan [9]. We further restricted our analysis to those targets whose mRNA transcripts have been found to interact with the miRNAs concerned in at least three independent CLIP-seq experiments [60]. We found that a number of relevant biological processes showed some enrichment in high-confidence targets of up-regulated miRNAs, most notably "cellular response to lipopolysaccharide" (S4 Table), while no enrichment was found in high-confidence targets of down-regulated miRNAs. To complement this analysis, we used miRNA and mRNA expression profiles from a previous study in the same cellular system upon MTB-Rv infection [55], to delineate mRNAs whose expression was significantly correlated with that of our core response miRNAs. Interestingly, mRNAs correlated with four core response miRNAs – miR-155–5p, miR-505–3p, miR-7-5p and miR-940 – showed an enrichment in innate immune functions (e.g. innate immune response, immune system process and response to bacterium) [55].

We next used hierarchical clustering to assess the correlation structure among core response miRNAs (Methods). Using the dynamic tree cut algorithm [61], we identified six clusters of highly correlated miRNAs (S3 Table). The two largest clusters contained 20 and 17 miRNAs, respectively. To identify upstream regulators that may explain the coexpression of miRNAs in

these two large clusters, we searched for an enrichment of transcription factor binding in the regions surrounding these miRNAs (Methods). Using transcriptional regulatory relationships from ChIP-seq data obtained from ChIPBase [62], we found that hairpins coding for core response miRNAs belonging to the largest of these clusters (i.e., cluster 1 in S3 Table) were most strongly enriched in the binding of the transcription factor MED12 in their promoter regions ($p = 4{\times}10^{-3}$). Interestingly, *MED12* has been found to be significantly upregulated upon MTB infection of DCs [63], suggesting a role of this gene in the regulation of this miRNA cluster in response to bacterial infection.

Distinct miRNA signatures in response to infection by virulent mycobacteria

Despite the generally strong similarity of miRNA responses to all bacteria in our panel (Fig. 1), closer examination revealed a number of more subtle signatures of variability that could be related to differences between bacteria. At the genome-wide level, hierarchical clustering based on miRNA expression levels indicated the presence of sub-clustering at each time point, with some bacteria showing more similar expression profiles following infection (Fig. 1B). This sub-clustering was further supported by a multidimensional scaling analysis (MDS) using the sum of edit distances calculated for each miRNA between bacteria based on their STEM model temporal response profiles (Methods). This showed a strong similarity between the responses to the two virulent MTBC bacteria (MTB-Rv and MTB-Bj), while the response to the attenuated BCG was intermediate between those of MTB-Rv and SLM (Fig. 2B). The separation of YRS in coordinate 1, indicating that the miRNA response to this bacterium was the most distinctive, may be due to the secretion of *Yersinia* outer proteins (Yops), which are unique to this bacterium and have previously been described to modulate host signalling pathways [64].

To further identify miRNA responses that were specific to virulent MTBC (vMTBC) bacteria, we fitted a generalized linear mixed model to test for the effect of infection with a vMTBC strain, while accounting for variability between infection conditions (Methods). We found that the magnitude of the response of 6, 5 and 14 miRNAs, at 4h, 18h and 48h respectively, was specific to infection with MTB-Rv and MTB-Bj (FDR-adjusted $p < 0.01$; S5 Table). This suggests that these miRNAs are part of a virulence-dependent response to mycobacterial infections.

Of the 20 miRNAs that showed a vMTBC-specific response, it is worth noting the presence of miR-132-3p, the only miRNA that was significant at all time points. This miRNA has previously been implicated in the regulation of the inflammatory response [65]. Additionally, both arms of miR-212—the other member of the miR-132/212 family due to their sequence homology and co-localisation on chromosome 17p13.3—also showed a vMTBC-specific response at 18h or 48h. To further investigate the role of virulence in the altered expression of the miR-132/212 family, we studied their response to an extended set of mycobacterial strains that differ in their virulence (Methods). Interestingly, we found that the attenuation and/or inactivation of MTB leads to a significantly lower induction of miR-132–3p, and to a lesser extent miR-212–3p, compared to virulent mycobacteria (Fig. 2C,D and S6 Table). Furthermore, infection with BCG::RD1 – a recombinant strain of BCG containing the RD1 locus, the absence of which accounts, to a large extent, for the attenuation of BCG [66] – significantly increased the induction of miR-132-3p and miR-212-3p, with respect to BCG, attaining a level that was not significantly different from cells infected with the virulent strains (Fig. 2C,D and S6 Table). Overall, these results indicate that the altered expression of the miR-132/miR-212 family is dependent on mycobacterial virulence and, more specifically, that the presence of the virulence-associated RD1 locus is sufficient to account for the stronger induction of the miR-132/212 family among virulent mycobacteria.

Infection induces changes in the relative expression of the arms of the miRNA hairpin

We next sought to move beyond considering miRNAs as single units to assess the impact of infection on other aspects of miRNA dynamics, from the broader context of the miRNA hairpin to the finer level of internal sequence variability. We first assessed whether infection induces changes in the relative expression of sequences derived from the 5p and 3p arms of the miRNA hairpin. In general, we found that one of the two arms is usually highly dominant. Of the 341 detected mature miRNAs that had a unique genomic alignment, 63% had only one arm expressed at detectable levels, of which one third had no annotated second sequence (Fig. 3A). Of the remaining 27%, 78% had at least a 10-fold dominance of one of the two arms, while only

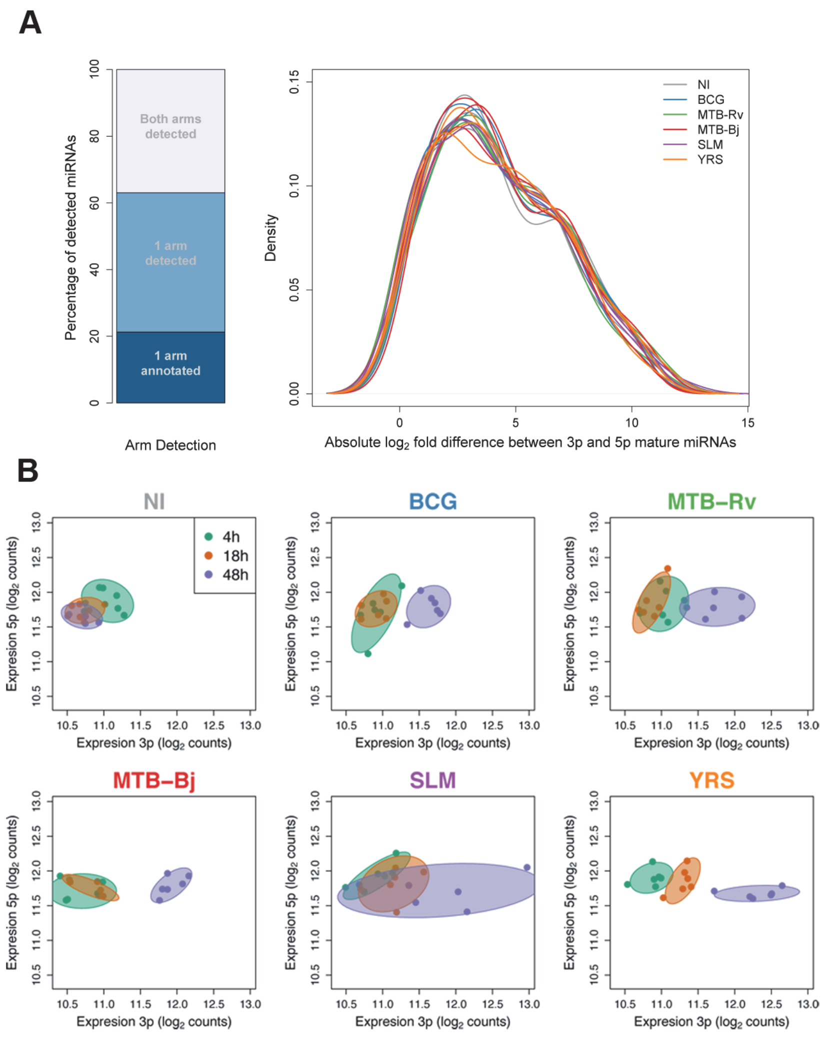

Fig 3. Changes in relative miRNA arm expression upon infection. (A) Detection and relative expression of miRNA hairpin arms. Stacked bar plot shows the proportion of detected miRNA hairpins for which either one or both mature miRNA sequences were detected. Density plot shows the distribution of log_2 expression ratios for hairpins where both arms were expressed. (B) An example of one miRNA, miR-361, which showed a strong infection-dependent change in relative arm expression. Axes show the expression of the 3p and 5p arms of all sequenced individuals at each time point. In each panel, colours denote the different time points, and infection conditions are plotted in separate panels. The displacement of points towards the right of the plot in infected samples at later time points shows that the change in the expression ratio of the two arms is due to the increased expression of the 3p arm that, in some conditions, becomes dominant.

doi:10.1371/journal.pgen.1005064.g003

15% showed less than a 2-fold difference between the expression levels of the two arms (Fig. 3A). These figures, however, are likely to underestimate the true dominance as we applied the same expression criteria for the detection of both arms of the hairpin.

We then examined the extent to which infection alters the relative expression of the arms of the miRNA hairpin for the 92 miRNAs for which both arms were detected (Methods). We detected 40 miRNAs that showed a significant change in relative arm expression in at least one experimental condition (FDR-corrected $p < 0.01$), with changes being broadly consistent, in tendency if not in statistical significance, across all bacteria (S7 Table). The majority of these changes grew in magnitude over time. Indeed, 13 hairpins showed differences in relative arm expression between time points in non-infected samples ($p<0.01$), suggesting that time also has a marked, albeit more modest, impact on this feature of miRNA abundance. Of the 27 hairpins (30% of those tested) for which the observed change in the relative expression of the hairpin arms was exclusively associated with infection, three of them (miR-199b, miR-361, miR-582) showed a change in the identity of the dominant miRNA arm upon infection with at least one bacterium (Fig. 3B, S7 Fig. and S7 Table). In most conditions, this change reflected the loss of arm dominance due to a change in the abundance of one of the two arms. However, in two cases (miR-361 and miR-582 expression following YRS infection for 48h) we observed a clear switch in the dominant miRNA sequence. To validate these observations, we measured the expression of one arm-switch miRNA – miR-361 – by qPCR, and obtained a highly concordant tendency of the change in relative expression upon infection (S6B Fig.).

Abundant changes in isomiR diversity in response to infection

We finally explored the internal sequence diversity of expressed miRNAs (i.e., isomiRs) at the steady-state and upon infection. In general, we detected much greater variability in the end site of isomiRs compared to their start positions (S8A Fig.). Moreover, this variability was dependent on the hairpin arm from which a miRNA was derived, with greater start-site variability among 3p miRNAs and greater end site variability in 5p miRNAs ($p = 3.39\times10^{-14}$ and $p = 3.15\times10^{-20}$, respectively; S8B Fig.). We next classified reads aligning to miRNAs into six groups according to their differences from the canonical miRNA sequence (Fig. 4A). We found that the canonical sequence was the dominant isomiR for only $\sim$50% of miRNAs, with the most common alteration being a change in the end position of the miRNA (3PC), a pattern that was consistently observed across conditions (Fig. 4B and S9 Fig.). Additionally, we observed high isomiR diversity for most miRNAs with the dominant read accounting for only half (median = 49.9%) of all reads aligning to a given miRNA (Fig. 4C).

To assess the impact of infection at the level of individual isomiRs, we searched for differentially expressed isomiRs using DESeq (see Methods) and detected 1,595 isomiRs, corresponding to 235 miRNAs, whose expression was altered upon infection (FDR-corrected p-value < 0.01 and $|\log_2 \text{fold change}| > 1$) (S8 Table). We found a significant overlap between these miRNAs and those that were differentially expressed at the level of total miRNA expression across experimental conditions (p-values = 1.15×10^{-4}–2.03×10^{-10}). However, we detected 132 additional miRNAs that show a response to infection at the isomiR level yet are missed when searching only for expression changes at the total miRNA level. Such changes may reflect (i) modest changes in the expression of one or a small number of isoforms that do not have a sufficiently strong cumulative effect on total miRNA levels to be detected, or (ii) changes in the relative proportions of isomiRs upon infection that do not result in an appreciable net gain or loss of reads aligning to the miRNA. When we tested for such changes in the relative expression of isomiRs in response to infection (see Methods), we identified 146 miRNAs that showed a significant change in isomiR proportions upon infection with at least one bacterium (S9

Table). Interestingly, although miRNAs showing changes in isomiR distribution upon infection showed a significant overlap with miRNAs that had one or more significantly differentially expressed isoforms (p-values = 2.48×10^{-5}–7.27×10^{-10}), we found only limited overlap between miRNAs showing changes in isomiR distribution and miRNAs that were differentially expressed at the whole miRNA level (p-values = 0.045–1), suggesting that this approach captures an additional aspect of miRNA variation in response to infection.

Changes in isomiR distribution increased over time and were strongly overlapping across bacteria, with only 22% unique to a single bacterial infection (Fig. 4D). For a subset of these

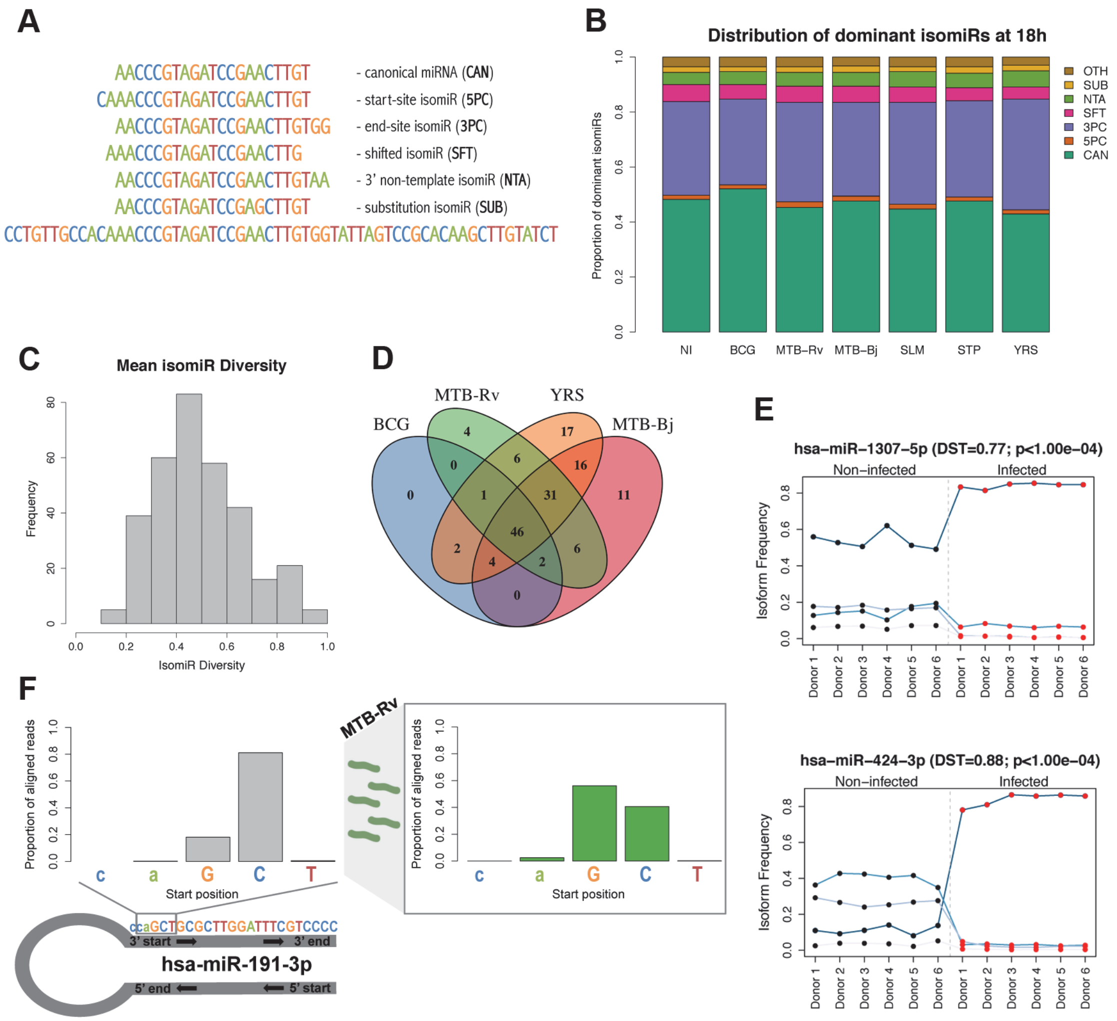

Fig 4. Dynamic expression of miRNA isomiRs upon infection. (A) Classification of isomiRs depending on their difference from the annotated mature miRNA sequence (based on [28]). (B) Cumulative barplots showing the proportion of miRNAs for which the dominant sequence was the canonical sequence or, instead, one of six classes of isomiR. Data shown is for 18h post-infection, for other time points see S9 Fig. (C) Histogram showing the mean miRNA diversity (expression of dominant isomiR / total expression of miRNA) across all experimental conditions. (D) Venn diagram showing the overlap of miRNAs showing a significant change in isomiR distribution upon infection with different bacteria. Due to the smaller sample size, we did not perform the analysis upon STP infection. We obtained no significant changes in isomiR distribution following SLM infection, as one individual did not respond to infection (S3 Fig.). (E) Examples of the two characteristic profiles observed for miRNAs showing a change in isomiR distribution upon infection; top: a difference in the proportional expression of each isomiR upon infection, bottom: a change in the dominant isomiR upon infection. Examples are taken from MTB-Rv infection at 48h. Each line represents an individual isomiR and the proportion of the total miRNA expression level accounted for by each isomiR, per donor, is shown before (black dots) and after (red dots) infection. Points are joined together for legibility. (F) The distribution of starting bases of reads aligning to miR-191-3p, which showed a seed shift upon infection with MTB-Rv, MTB-Bj and YRS. Before infection (left), less than 20% of reads start at the first "G" position of the canonical sequence, whereas after infection (right), over 50% of reads start at this position. Proportions of aligned reads shown in the figure are following MTB-Rv infection at 48h.

doi:10.1371/journal.pgen.1005064.g004

miRNAs, this change in isomiR distribution involved a change in the dominant isomiR sequence between non-infected and infected samples (Fig. 4E and S10 Table). For those miRNAs where the canonical miRNA was the dominant sequence in one of the two conditions (~70% of cases), a switch from the canonical sequence before infection to a non-canonical isoform upon infection was 3× more common than the reverse trend. Consistent with the expression levels of different isomiR classes, the majority of dominant isomiR switches (77–100% per condition) involved a change in the 3' terminus of the most abundant sequence. Interestingly, we identified seven miRNAs – miR-98-3p, miR-140–3p, miR-191-3p, miR-342-5p, miR-548e-5p and miR-2116–3p – that showed a change in the 5' start site of the dominant sequence upon infection with one or more bacteria (Fig. 4F).

To assess the impact of a change in the 5' start site, and hence the functional seed sequence, on potential miRNA targets, we used TargetScan's custom target prediction [67]. We found that 45–87% (mean 71%) of the predicted targets of the annotated miRNA sequence are not predicted targets of the alternative 5' shift isomiR, suggesting that these isomiR changes could profoundly impact upon miRNA-mediated functions. In addition, that miRNAs showing changes in isomiR distribution in response to infection were highly expressed compared to genome-wide miRNA expression levels (Mann-Whitney p-values 8.13×10^{-4}–2.88×10^{-9}) suggests that these changes are sufficiently highly abundant to be of functional relevance. These results, together with our observation that infection can also lead to changes in the relative abundance of the hairpin arms, indicate an even greater dynamism in the regulation of miRNA expression than previously appreciated.

Discussion

In this study, we have shown that the miRNA repertoire involved in the host cellular response to infection is highly similar across a set of different bacteria, both qualitatively – in the identities of differentially expressed miRNAs – and quantitatively – in the high concordance of their expression dynamics upon infection and over time (Fig. 1 and Fig. 2). Specifically, we found that less than 10% of differentially expressed miRNAs are unique to a single bacterium, and defined a set of 49 miRNAs – one third of all differentially expressed miRNAs – that characterises a core response to bacterial infection. Such temporally conserved miRNA responses across bacteria, despite their genetic diversity and differing strategies to manipulate host cell functions [68–70], most likely reflect the activation of common or convergent signalling pathways in response to infection, as has been shown for mRNAs [3,71,72]. For example, the expression of two core response miRNAs—miR-155-5p and let-7i-3p – is known to be induced by the activation of TLRs, key innate immunity receptors that recognise a diverse array of microbial products and the signalling of which is regulated, in part, by miRNAs [73,74].

The consistent changes observed among core miRNAs upon infection raises the question of whether such changes are essential for establishing and maintaining an effective immune response to infection. Though the importance of a small number of miRNAs for the immune response has been described [12,74–76], our understanding of the roles played by miRNAs in the regulation of this and other biological processes remains limited. Some insight can be gained from the study of computationally predicted miRNA targets, however the limited complementarity of animal miRNAs and target sites makes this challenging [7,8]. Additionally, such computational tools are restricted by our current knowledge of the rules of miRNA targeting and do not account for cell-specific interactions, limitations that are reflected in the high false positive and false negative rates of such algorithms [77]. Targets have also been identified through a range of experimental approaches, each of which carry their own limitations [78], and, critically, few targets have been functionally validated. Despite these limitations, the

enrichment analyses of both predicted targets and correlated mRNAs, as well as existing knowledge of the function of some miRNAs, point to the involvement of our set of core response miRNAs in the innate immune response. This suggests that this miRNA response plays a genuine role in the regulation of basic cellular responses to stress, at least in DCs, rather than being a side effect of the immunological changes following infection.

Our results also revealed some important elements of variability in miRNA transcriptional responses between bacteria that may provide insight into bacterial pathogenesis. In this context, we demonstrated that the induction of the miR-132/212 family characterises the response to virulent mycobacteria and is dependent on the presence of the virulence-associated RD1 locus (Fig. 2C,D). Moreover, the reduced induction of miR-132/212 after infection with an attenuated strain that secretes lower levels of the RD1-encoded virulence effector protein ESAT-6 [79] indicates that such an induction is associated with the secretion of this virulence factor. These results raise questions regarding the underlying mechanisms and potential functional consequences of this response, and of the other virulence-dependent miRNA signatures we identified. For example, in light of the role of miR-132 in the negative regulation of the inflammatory response [65,74,80], it is tempting to speculate that the stronger miR-132 induction we observe contributes to the dampening of the early inflammatory response to infection by virulent mycobacteria. Interestingly, reduced early inflammatory responses have been observed in response to modern MTBC lineages, which include MTB-Rv and MTB-Bj, and have been associated with faster progression and increased virulence in macrophages [81]. Further experimental work is now needed to substantiate this hypothesis and, more generally, to identify the specific mycobacterial virulence factors associated with host miRNA expression dynamics.

One of the most interesting findings of our study, made possible by the use of sRNA-seq, is that infection induces changes in both the relative expression of the arms of the miRNA duplex and the distribution of isomiRs (Fig. 3 and Fig. 4). In particular, the induction of strong changes in isomiR distributions, which were highly concordant across individuals and bacteria, highlights the dynamism of miRNA biogenesis and raises important questions regarding the regulation of this process. Several features of our results strongly support that these sequence variants represent true isomiRs showing genuine, infection-dependent changes in their distribution and expression. The isomiRs that we report are expressed at appreciable frequencies and involve more frequent changes at the 3' end of the sequence (S8A Fig.), consistent with the conservation of the seed region [82], which are concordant with known post-transcriptional modifications of miRNAs, such as the non-template addition of, exclusively, "A" and "T" nucleotides [83,84]. In addition, the inverse differences in start and end site variability between miRNAs derived from 3p and 5p arms of the miRNA hairpin (S8B Fig.), supports a greater specificity of Drosha, compared to Dicer, cleavage, as recently suggested [37,41]. More importantly, although specific sequence variants could, theoretically, be the result of errors in the trimming of the sequencing adaptors, these would be expected to occur systematically across conditions and cannot therefore account for the reproducible differences in isomiR expression and/or proportions observed between non-infected and infected cells.

Our results suggest that infection, or the cellular response it elicits, alters one or more of the cellular processes that regulate miRNA expression and isomiR production. The control of miRNA homeostasis is a highly complex and dynamic process involving the transcriptional and post-transcriptional regulation of miRNA expression, biogenesis, loading and decay [19,23]. Even a slight disruption of any of these highly integrated stages could have profound yet variable consequences for miRNA abundance and isomiR diversity as well as, potentially, miRNA functions. For example, isomiR-generating post-transcriptional modifications such as nucleolytic trimming and 3' uridylation and adenylation have been associated with changes in

miRNA stability, loading into the miRISC complex and target gene expression [32,35,83–86], and some viruses have been shown to interfere with these processes [87].

We also found that some miRNAs, including miR-191-3p and miR-342-5p, show a seed shift upon infection. Seed shift isomiRs can have distinct, though overlapping, sets of target genes [28,84,88], as highlighted by our results that show a modest 30% overlap between shifted and canonical sequences, suggesting that the targeting properties of these miRNAs are altered upon infection. More broadly, that the majority of changes at the isomiR level were not detected at the whole miRNA level highlights that focusing only on total miRNA expression misses potentially important changes in miRNA regulation. However, it should be kept in mind that ~85% of all miRNAs had at least one differentially expressed isomiR, emphasising the need to understand further how much of this variability is tolerated by the cell without any biological impact on gene regulation.

In conclusion, our study has reported extensive changes in miRNA expression upon infection that are highly concordant across diverse bacteria and over time. Our results represent the most comprehensive, unbiased view of the similarities in miRNA responses between bacteria to date, and highlight common miRNA-mediated mechanisms that are likely to be essential in the cellular response to stress. Conversely, the detected differences between bacteria may reflect more subtle variations in magnitude and tempo that could, nonetheless, impact on bacterial pathogenesis, such as the case of the induction miR-132-3p. Overall, our findings highlight a novel aspect of miRNA expression dynamics upon infection and increase our understanding of miRNA-mediated mechanisms involved in host cellular responses to infection. In doing so, they provide new perspectives concerning the ways in which infection leads to changes in cellular processes that regulate miRNA expression and isomiR production.

Materials and Methods

Ethics statement

Blood samples from nine healthy donors were obtained from the Etablissement Français du Sang. Signed, written consent was obtained from all individuals. The biobank has been declared to and recorded by both the French Ministry of Research and the French Ethics Committee under the reference DC-2008-68 collection 2.

Bacterial preparation

We infected DCs from six individuals with a panel of six bacteria comprised of: two strains of *Mycobacterium tuberculosis* (H37Rv and GC1237), *Mycobacterium bovis*-BCG Pasteur, *Salmonella typhimurium* strain Keller, *Yersinia pseudotuberculosis* and *Staphylococcus epidermidis* (MTB-Rv, MTB-Bj, BCG, SLM, YRS and STP, respectively). Mycobacteria were grown from a frozen stock to mid-log phase in 7H9 medium supplemented with albumin-dextrose-catalase (Difco). Liquid cultures were grown for up to 12 days and stored at −80°C in 1–2ml aliquots with 10% glycerol. Aliquots were thawed 1 week before infection and bacteria were grown to mid-log phase. Before infection, bacteria were washed 2 times with and re-suspended in 1ml of PBS. Mycobacterial clumps were disassociated by passages through a needle, followed by 5 minutes of sedimentation. Clinical isolates of SLM, STP and YRS were grown on Luria-Bertani agar and stored at −80°C. One day before infection, aliquots were thawed and bacteria grown overnight. 1ml of bacterial culture was grown to mid-log phase shortly prior to infection. Bacterial density in the supernatants was checked at OD600 and confirmed by counting colony-forming units. We infected DCs from three additional individuals with a second panel of six MTBC bacteria comprised of: BCG transformed with the empty cosmid pYUB (BCG) or the same cosmid containing RD1 (BCG::RD1), *Mycobacterium tuberculosis* H37Ra (MTB-Ra), a

non-virulent strain of MTB, heat-killed *Mycobacterium tuberculosis* H37Rv (MTB-Hk), MTB-Rv and MTB-Bj. All strains were prepared as described above for mycobacteria.

Isolation and infection of DCs

Blood mononuclear cells were isolated by Ficoll-Paque centrifugation. Monocytes were purified from peripheral blood mononuclear cells by positive selection with magnetic CD14 MicroBeads (Miltenyi Biotech). Monocytes were then cultured for 5 days in RPMI-1640 (Invitrogen) supplemented with 10% heat-inactivated FCS (Dutscher), L-glutamine (Invitrogen), GM-CSF (20 ng/mL; Immunotools), and IL-4 (20 ng/mL; Immunotools). Cell cultures were fed every 2 days with complete medium supplemented with the cytokines previously mentioned. The resulting monocyte-derived DCs were infected ($\sim 2{\times}10^6$ cells per condition) at an MOI of 1:1 with one of the bacterial panel, or left uninfected, for 1h at 37°C. The cells infected with bacteria of the first panel, or left uninfected, were washed and cultured for a further hour with 50μg/ml gentamycin. The cells were then washed a second time and cultured in complete medium with 5μg/ml gentamycin for an additional 4h, 18h and 48h. In total, we assessed 21 conditions per individual: seven infection conditions (six bacterial infections plus non-infected cells) at three different time points. Due to material limitations and the proliferation rate of the bacteria, we were only able to perform infections and/or recover cells for four of the six individuals and only at 4h and 18h after infection with STP, giving a final total of 116 samples. The cells infected with bacteria of the second panel, or left uninfected, were cultured in complete medium, without gentamycin, for an additional 18h.

Library preparation and sequencing

Total RNA was extracted using the miRNeasy kit (Qiagen). RNA quantity was assessed using the Qubit (Life Technologies) and RNA quality was assessed using the Agilent 2100 Bioanalyzer with the Nano chip (Agilent Technologies). All samples were of very high quality and showed no signs of degradation (mean RNA integrity number = 9). Sequencing libraries were prepared for each of the 116 samples using the Illumina TruSeq protocol following isolation of low molecular weight (small) RNA fragments. Once prepared, indexed cDNA libraries were pooled (8 or 12 libraries per pool) in equimolar amounts and sequenced with single-end 50bp reads on the Illumina HiSeq2000.

Pre-processing of raw sequencing reads

Raw reads were first assigned to individual samples based on their multiplexing index, allowing for 1 mismatch. We obtained an average of 11.9 million raw reads per sample with a minimum yield of 6.3 million reads. Next, sequences matching the 3' adaptor sequence were identified and trimmed. A minimum matching of the 6 first bases of the adaptor sequence was required giving reads with final real lengths of 0 to 44 bases. Sequence quality was assessed and subsequent processing performed in R using the Bioconductor package ShortRead [89]. Specifically, we confirmed that base quality (Q) values and per-base GC distributions were within expected ranges and that read length distributions showed an enrichment of reads of the same length as mammalian miRNAs ($\sim$22 bases) (S10 Fig.). We further removed repetitive and low complexity reads. Specifically, we discarded all reads that contained a mononucleotide repeat longer than 10 bases, and those that were >75% mono-, di- or tri-nucleotide repeat or >20% "N" bases. Lastly, we discarded all reads shorter than 16 or longer than 26 bases, corresponding to the length distribution of mammalian miRNAs. After these filtering steps, we obtained an average of 9.1 million (minimum 3.8 million) clean, short reads per sample that were used for small RNA quantification.

Sequence alignment

Sequences were aligned to the human reference genome (build GRCh37/hg19) using bowtie (version 0.12.7) [90]. We mapped reads allowing for 2 mismatches (-v 2) and reported all best alignments for reads that mapped equally well to more than one genomic location (-a —best —strata). We suppressed reads with more than 50 possible alignments (-m 50). On average, 98% of reads aligned to the human genome, of which 65% aligned uniquely. As miRNAs are short and tend to occur in families that share highly similar sequences, they are particularly susceptible to spurious multiple alignments, a phenomenon called cross-mapping. Around 35% of reads in the present dataset had more than one best alignment. To avoid cross-mapping artefacts, we used a correction strategy that assigns weights to each of the candidate mapping loci of multiply aligning reads [91]. Weights were calculated based on local expression levels and mismatches in the alignment. Python scripts were obtained from the authors and implemented, without modification, as described in the original manuscript [91].

Prediction of novel miRNAs

We used the program miRDeep2 [56] to detect putative novel miRNAs in our data using a two-step approach. First, we used the mapper module to map all reads to the human reference genome (build GRCh37/hg19) using bowtie (version 0.12.7) with default miRDeep2 alignment parameters [56]. We ran the module on fastq files from all 116 samples by specifying a config file containing a unique identifier for each sample. We removed all sequences containing a base other than A, C, T, G, U or N, collapsed identical reads and output the pooled dataset in fasta format (mapper.pl –d –e –j –h –m –p). We then used the miRDeep2 module to identify novel and known miRNAs in the pooled set of aligned reads from all 116 samples. All reference files containing either mature or precursor sequences of known miRNAs were from miRBase v20, thus we used the –P flag to specify that miRBase identifiers are in post-v18 "5p" and "3p" format. We considered *Pan troglodytes*, *Pan paniscus*, *Gorilla gorilla* and *Pongo pygmaeus* as related species, as described in the miRDeep2 paper [56]. We validated the miRDeep2 mapping and quantification algorithms by comparing read counts of known miRNAs with our own and confirmed that these were highly correlated. We defined a set of high-confidence putative novel miRNAs using a miRDeep score cut-off of 4 (S2 Fig.). We further removed those predictions that overlapped protein-coding exons, based on Ensembl v75 annotations (www.ensembl.org), as well as those that had a predicted hairpin length less than 45 bases or for which no complementary (star) miRNA was detected.

Expression analysis of annotated and novel miRNA transcripts

We extracted reads aligning to annotated mature miRNA sequences (miRBase v20) or our high confidence set of putative novel miRNAs with at least 75% overlap using BEDTools [92]. As we had libraries that were sequenced to different depths, we normalised the data to give comparable numbers of reads for each sample. Specifically, we used DESeq (version 1.10.1) to calculate a size factor for each library and divided read counts by this factor [57]. To remove lowly or sporadically expressed miRNAs, we kept only those miRNAs with scaled counts of greater than 50 reads in at least three samples from at least one experimental condition.

We used DESeq (version 1.10.1) to identify differentially expressed miRNAs upon infection by fitting a generalized linear model using a negative binomial distribution [57]. Specifically, for each experimental condition we compared miRNA expression levels between non-infected and infected samples at the same time point. As non-infected and infected samples came from the same six donors (four in the case of STP), we controlled for the paired nature of our data by specifying donor identity in our model. We corrected for multiple testing using a stratified

false discovery approach, taking a per-condition Benjamini and Hochberg FDR-corrected p-value < 0.01. We also required an absolute $\log_2$ fold change greater than 1. We performed unsupervised hierarchical clustering on the 50 most variable miRNAs (i.e. those with the highest variance in expression across all samples) using mean miRNA expression levels, after variance stabilisation, with the *heatmap.2* function of the R package gplots.

To test for cases where the miRNA response was specific to virulent MTBC bacteria (MTB-Rv and MTB-Bj), while accounting for variability between conditions, we used the R package glmmADMB [93]. We fit a generalized linear mixed model assuming a negative binomial distribution of miRNA expression:

$$\log(\mu_{ik}) = a + b\mathbf{1}_{Infected_k} + c\mathbf{1}_{MTB_k} + d_k$$

where μ_{ik} is the mean read count for individual i in experimental condition k (bacterial strain or non-infected), $\mathbf{1}_{Infected_k}$ is a categorical variable indicating the presence of infection in condition k (irrespective of the bacterial strain), $\mathbf{1}_{MTB_k}$ is a categorical variable indicating the presence of infection with a virulent MTBC bacterium in condition k, $d_k \sim \mathrm{N}(0,\sigma_k^2)$ is a random effect of condition k, and where a, b and c are all fixed effects to be estimated from the model. We corrected for multiple testing using a stratified false discovery approach, taking a per-condition Benjamini and Hochberg FDR-corrected p-value < 0.01.

We also compared, for annotated miRNAs only, the relative expression level of the 5p and 3p arms of the miRNA duplex between experimental conditions. To do so, we selected only miRNAs where both arms of the hairpin were expressed, had a unique genomic location and did not contain a known polymorphism in their mature sequence (MAF>1% in the European CEU population from the 1,000 Genomes Project [94]). We next calculated, for each sample and miRNA hairpin, the ratio between the expression levels of 5p and 3p mature miRNAs ($\log_2$(5p read count / 3p read count)). We then used a paired, two-sided t-test to test for a significant change in this ratio between non-infected and infected samples at the same time point. We used a cut-off of a per-condition Benjamini and Hochberg FDR-corrected p-value < 0.01 to determine significance. To assess the effect of time on the relative expression of 5p and 3p-derived miRNAs, we performed the same analysis comparing, pairwise, non-infected samples at the three time points.

Expression analysis of isomiRs

We extracted reads aligning to annotated mature miRNA sequences as described above and directly normalised the read counts using the same approach as applied for total miRNA expression levels. We removed all reads aligning to miRNAs with multiple genomic alignments and miRNAs that contained a SNP (MAF>1% in the European CEU population from the 1,000 Genomes Project [94]) in their mature sequence. We considered each unique read as a potential miRNA isoform (isomiR). We classified isoforms into six categories: (i) canonical sequences (according to miRBase v20); (ii) changes in start site; (iii) changes in end site; (iv) shifted sequences; (v) containing a substitution; and (vi) non-templated 3' additions; as well as a seventh mixed group of reads containing multiple types of change. We identified isomiRs that were differentially expressed upon infection using the same filtering criteria, approach and significance thresholds as described for total miRNA expression levels. To assess the impact of infection on the distribution of isoforms for a given miRNA, we defined a statistic (D_{ST}) that estimates differentiation in isoform proportions between populations of samples. D_{ST} is analogous to F_{ST} [95,96] and V_{ST} [97], which are also used for detecting population differentiation.

D_{ST} varies between 0 and 1 and is calculated by considering,

$$D_{ST} = \frac{D_{all} - (D_a + D_b)/2}{D_{all}}$$

where D_a is the mean Euclidean distance between isomiR proportions of samples from condition *a*, D_b is the mean distance between samples from condition *b*, and D_{all} is the mean distance between samples across conditions. Distances were calculated using the R function *dist* on the proportions of all detected isomiRs for a given miRNA. We calculated p-values based on 10,000 permutations of isomiR proportions, with replacement, per miRNA. We used a cut-off of an empirical p-value < 0.001 to determine significance. We found that 97% of the significant changes identified by D_{ST} were also detected using AMOVA [95], in the same experimental condition and using the same statistical cut-off, confirming that our metric captures relevant changes in isomiR distribution.

Temporal expression profiles of miRNAs in response to infection

We used the Short Time-series Expression Miner (STEM) to characterise the miRNA responses to each bacterium over time [58,59]. This software assigns observations to a pre-determined set of model temporal response profiles based on the correlation coefficient between observed data (i.e., fold-change at each time point) and model profiles. We used default settings except for the maximum unit change between sequential conditions, which we restricted to 1. To account for inter-individual variability, we simultaneously analysed miRNA expression data for all profiled individuals in a given condition using the "repeat data" option. As we did not measure miRNA expression at 0h, we used the "no normalization / add 0" option to set this value to 0. Fold changes were thus calculated by comparing expression levels before and after infection at the same time-point. To account for the fact that STEM allocates a miRNA to a single model profile, even though it may show a strong correlation with one or more additional profiles, we merged clusters where the model profiles were strongly correlated with each other ($r>0.8$). We defined the core miRNA response on the basis of these merged model profiles.

We further used these, STEM-assigned, model profiles to calculate edit distances between pairs of bacteria for a given miRNA based on the number of steps required to change between their respective model profiles. For example, the edit distance between the profile 0,0,1 and 0,1,2 would be 2 while the distance between 0,-1,-1 and 0,-1,-2 would be 1. We used the sum of these pairwise edit distances to represent the difference between a given pair of bacteria in terms of their miRNA response and visualised this by nonmetric multidimensional scaling using the R function *isoMDS* from the MASS package.

Enrichment of predicted miRNA targets in Gene Ontology categories

We identified high-confidence predicted miRNA targets by combinbing TargetScan target predictions [9,67,98] and miRNA-protein interaction data based on CLIP-seq using the StarBase database of high-confidence interactions [60]. Gene Ontology (GO) biological processes were downloaded from the website of the Gene Ontology Consortium (http://geneontology.org/). We first checked that core response miRNAs were not significantly different from all other miRNAs with respect to their conservation, GC content, and number of predicted targets. We then calculated the proportion of high-confidence predicted targets of core miRNAs in each GO category. Next, we calculated the same measure for 10,000 randomly resampled size-matched miRNA sets and used this to calculate an enrichment p-value. This p-value reflects

the fraction of random miRNA sets having a greater proportion of predicted targets in a given GO category compared to the test set.

Analysis of miRNA coexpression

We performed hierarchical clustering on miRNA expression levels using the package wgcna with default settings [99,100]. Specifically, we used the dissimilarity of the Topographical Overlap Matrix and average linkage to cluster the 49 core response miRNAs based on normalised miRNA expression levels across all 116 samples. We then used the dynamic tree cut method to cut the branches of the dendrogram to give clusters of highly correlated miRNAs [61]. We used the ChIP-Base database to identify transcription factors (TFs) that bind to miRNA promoter regions, defined as the region from 5kb upstream to 1kb downstream of the transcription start site of the miRNA [62]. As this database contains results from many different tissues and cell lines, we only considered whether binding had been detected, or not, in the promoter region. To identify TFs that were significantly more frequently bound close to coexpressed miRNAs than expected, we compared the average number of bound factors per miRNA to 10,000 randomly sampled size-matched miRNA sets.

Real-time quantification of miRNAs

Total RNA was extracted using the miRNeasy kit (Qiagen). To quantify miRNA expression levels, cDNA was synthesized and quantitative real-time PCR (qPCR) performed using the Qiagen miScript PCR system and primers (miScript II RT Kit: 218161; miScript SYBR Green PCR kit: 218073; miR-92b-3p MS00032144; miR-132-3p MS00003458; miR-155–5p MS00031486; miR-212-3p MS00003815; miR-361-5p MS00004032; miR-361-3p MS00009555; U6 MS00033740) in a 7900 Real-time PCR system (Applied Biosystem). Relative miRNA expression levels, normalized to the endogenous control U6, were calculated using the $\Delta\Delta C_t$ method [101].

Supporting Information

S1 Fig. General workflow and study design. (A) Experimental conditions used and (B) small RNA sequencing data analysis workflow.
(TIF)

S2 Fig. Detection of putative novel miRNAs. (A) Quality control of miRDeep2 novel miRNA discovery using pooled data from all 116 samples. (B) Example of a high confidence putative novel miRNA identified by our approach.
(TIF)

S3 Fig. Principal Component (PC) analysis of miRNA expression levels. PC analysis was performed on the normalised read counts of 405 robustly expressed miRNAs reported in the results section of this paper. Components were calculated using the R function *prcomp*, on centred and scaled data. The similarity of the distribution of PCs to known biological variables and potential technical confounders was calculated using a Kruskal-Wallis rank sum test, with the R function *kruskal.test*. We performed the analysis on all samples together (n = 116) and separately on samples from each of our three time points (4h, 18h and 48h). We found that length of infection accounted for the greatest amount of variance between all samples, consistent with our observation of the increase in the number of differentially expressed miRNAs over time and the results of our clustering analysis (Fig. 1A,B). Consistently, when we repeated PCA on samples from each time point separately we observed that the bacteria with which the samples were infected account for an increasing proportion of the variance with time. Specifically, we

observe no correlation of infection with any of the 10 first PCs at 4h, a significant correlation with PC5 at 18h and a significant correlation with PC1 after 48h. We also found, when considering all time points together, a significant correlation between the individual donors and PCs 3 and 4, suggesting a meaningful amount of interindividual variability in miRNA expression. However, we observed no correlation between the PCs and technical variables such as sequencing run, sequencing lane and index sequence, suggesting that these potential technical confounders do not substantially influence our results.
(TIF)

S4 Fig. Correlation of $\log_2$ fold changes of miRNAs in response to infection between different bacteria at different time points. We observed a strong correlation in the fold changes of miRNAs upon infection with our panel of diverse bacteria at 18h and 48h. The correlation was less pronounced at 4h for many comparisons, an observation that may be partially accounted for by the globally smaller fold changes at this early time point.
(TIF)

S5 Fig. Core temporal responses of novel miRNAs. Two putative novel miRNAs (chr6_41188-5p and chr19_18208-5p) that showed highly concordant changes in expression profiles over time across all bacteria. As no expression profiles were available for the 48h time point for STP infection, this bacterium was excluded from the analysis.
(TIF)

S6 Fig. Validation of core miRNA and arm-switch miRNA expression profiles by qPCR. (A) Response to infection of two core miRNAs (miR-155-5p and miR-92b-3p) measured by qPCR. We assessed their induction upon infection with each of our bacteria compared to non-infected cells at the same time point. We confirmed that these miRNAs were significantly induced upon infection with all bacteria at both 18h and 48h. For miR-155-5p, the fold changes were more pronounced at 48h than at 18h, while for miR-92b-3p fold changes were broadly consistent for each bacterium between the two time points, consistent with the temporal induction profiles observed for each by sRNA-seq (Fig. 2A and S3 Table). (B) Relative expression of the arms of the mir-361 hairpin measured by qPCR. We compared their relative expression before and after infection at 18h and 48h. Consistent with our sRNA-seq results, we observed a change in the ratio of the expression of the two arms upon infection. This change was most pronounced at 48h and resulted in a switch in the dominant arm of the miRNA following YRS infection. It should be noted that the fold changes observed by qPCR are much smaller than those obtained by sRNA-seq. This is most likely due to the different way in which expression is measured and calibrated by qPCR, where higher expression is denoted by a lower CT value and normalised to the expression of a housekeeping gene. Importantly, however, the tendencies we observed by sRNA-seq remain the same. The data presented in both panels represent the mean of a duplicate of qPCR calculated for four independent donors (of the six profiled by sRNA-seq). Expression levels were normalised on RNU6-1. The additional two donors as well as the 4h time point were excluded due to limited sample availability.
(TIF)

S7 Fig. Relative arm expression of three miRNAs showing a change in the dominant arm of the hairpin upon infection with at leas one bacterium. Significance, compared to the non-infected condition, was calculated using a paired t-test (** = FDR-adjusted $p < 0.01$).
(TIF)

S8 Fig. Variability around the start and end site of annotated miRNAs. (A) Greater variability was observed at the end of the miRNA sequence, relative to the end-point of the canonical

sequence, compared to the start. Specifically, while over 95% of reads aligning to a given miRNA had the same start position as that of the canonical sequence, less than 60% of aligned reads had the same end position as the canonical sequence, with extensions compared to the canonical position more common than shortening. (B) The extent of variability at the start and end sites was differentially distributed between mature miRNAs derived from the 3p and 5p arms of the miRNA hairpin. Specifically, a lower proportion of reads aligning to 3p miRNAs shared the canonical start site compared to reads aligning to 5p miRNAs. Conversely, a lower proportion of reads aligning to 5p miRNAs shared the canonical end site compared to reads aligning to 3p miRNAs. In other words, 3p-derived miRNAs showed relatively greater start-site variability while 5p miRNAs showed relatively greater end-site variability.
(TIF)

S9 Fig. Proportions of dominant isomiR classes. Cumulative barplots showing the proportion of miRNAs for which the dominant sequence was the canonical sequence or, instead, one of six classes of isomiR at 4h and 48h. The results for the 18h time point are presented in Fig. 4B, and a schematic representation of the isomiR acronyms given in the legend is presented in Fig. 4A.
(TIF)

S10 Fig. Quality control of Illumina sequencing data. (A) Base quality, (B) GC content distribution and (C) insert lengths of raw sequence reads. Values shown are for one representative sequencing run of 18 samples. All samples were systematically randomised for sequencing to avoid technical confounders that could prevent the detection of true differences between experimental conditions.
(TIF)

S1 Table. List of expressed miRNAs with p-values and fold-changes of their differential expression upon infection compared to non-infected samples
(XLSX)

S2 Table. Genomic locations of novel miRNA hairpins described in the study.
(XLSX)

S3 Table. STEM-assigned model temporal response profiles and wgcna clusters for core response miRNAs.
(XLSX)

S4 Table. Fifty most enriched Gene Ontology biological processes among high-confidence predicted targets of up-regulated core response miRNAs.
(XLSX)

S5 Table. List of expressed miRNAs with p-values of the specificity of their response following infection with virulent MTBC bacteria.
(XLSX)

S6 Table. Differential expression of the miR-132/212 family following infection with the extended MTBC panel.
(XLSX)

S7 Table. miRNAs showing significant changes in relative arm expression upon infection.
(XLSX)

S8 Table. List of expressed isomiRs with p-values and fold-changes of their differential expression upon infection compared to non-infected samples.
(XLSX)

S9 Table. List of expressed miRNAs with p-values and D_{ST} statistics reflecting infection-induced changes in isomiR distribution.
(XLSX)

S10 Table. List of the dominant isomiR in each experimental condition.
(XLSX)

Acknowledgments

We thank Roland Brosch for the generous donation of recombinant bacterial strains and Maxime Rotival, Guillaume Laval, Frederik Gwinner and Robin Friedman for helpful discussions.

Author Contributions

Conceived and designed the experiments: KJS LT LQM. Performed the experiments: KJS LT MD CD LF. Analyzed the data: KJS. Contributed reagents/materials/analysis tools: YHEL BG CA LBB. Wrote the paper: KJS LQM.

References

1. Chaussabel D, Semnani RT, McDowell MA, Sacks D, Sher A, et al. Unique gene expression profiles of human macrophages and dendritic cells to phylogenetically distinct parasites. Blood 2003; 102: 672–681. PMID: 12663451
2. Huang Q, Liu D, Majewski P, Schulte LC, Korn JM, et al. The plasticity of dendritic cell responses to pathogens and their components. Science 2001; 294: 870–875. PMID: 11679675
3. Chevrier N, Mertins P, Artyomov MN, Shalek AK, Iannacone M, et al. Systematic discovery of TLR signaling components delineates viral-sensing circuits. Cell 2011; 147: 853–867. doi: 10.1016/j.cell.2011.10.022 PMID: 22078882
4. Fairfax BP, Humburg P, Makino S, Naranbhai V, Wong D, et al. Innate immune activity conditions the effect of regulatory variants upon monocyte gene expression. Science 2014; 343: 1246949. doi: 10.1126/science.1246949 PMID: 24604202
5. Gat-Viks I, Chevrier N, Wilentzik R, Eisenhaure T, Raychowdhury R, et al. Deciphering molecular circuits from genetic variation underlying transcriptional responsiveness to stimuli. Nat Biotechnol 2013; 31: 342–349. doi: 10.1038/nbt.2519 PMID: 23503680
6. Lee MN, Ye C, Villani AC, Raj T, Li W, et al. Common genetic variants modulate pathogen-sensing responses in human dendritic cells. Science 2014; 343: 1246980. doi: 10.1126/science.1246980 PMID: 24604203
7. Bartel DP. MicroRNAs: genomics, biogenesis, mechanism, and function. Cell 2004; 116: 281–297. PMID: 14744438
8. Huntzinger E, Izaurralde E. Gene silencing by microRNAs: contributions of translational repression and mRNA decay. Nat Rev Genet 2011; 12: 99–110. doi: 10.1038/nrg2936 PMID: 21245828
9. Friedman RC, Farh KK, Burge CB, Bartel DP. Most mammalian mRNAs are conserved targets of microRNAs. Genome Res 2009; 19: 92–105. doi: 10.1101/gr.082701.108 PMID: 18955434
10. Chen CZ, Li L, Lodish HF, Bartel DP. MicroRNAs modulate hematopoietic lineage differentiation. Science 2004; 303: 83–86. PMID: 14657504
11. Johnnidis JB, Harris MH, Wheeler RT, Stehling-Sun S, Lam MH, et al. Regulation of progenitor cell proliferation and granulocyte function by microRNA-223. Nature 2008; 451: 1125–1129. doi: 10.1038/nature06607 PMID: 18278031
12. O'Connell RM, Rao DS, Baltimore D. microRNA regulation of inflammatory responses. Annu Rev Immunol 2012; 30: 295–312. doi: 10.1146/annurev-immunol-020711-075013 PMID: 22224773
13. Lodish HF, Zhou B, Liu G, Chen CZ. Micromanagement of the immune system by microRNAs. Nat Rev Immunol 2008; 8: 120–130. doi: 10.1038/nri2252 PMID: 18204468

14. O'Connell RM, Rao DS, Chaudhuri AA, Baltimore D. Physiological and pathological roles for microRNAs in the immune system. Nat Rev Immunol 2010; 10: 111–122. doi: 10.1038/nri2708 PMID: 20098459

15. Cullen BR. Viruses and microRNAs: RISCy interactions with serious consequences. Genes Dev 2011; 25: 1881–1894. doi: 10.1101/gad.17352611 PMID: 21896651

16. Eulalio A, Schulte L, Vogel J. The mammalian microRNA response to bacterial infections. RNA Biol 2012; 9: 742–750. doi: 10.4161/rna.20018 PMID: 22664920

17. Calin GA, Croce CM. MicroRNA signatures in human cancers. Nat Rev Cancer 2006; 6: 857–866. PMID: 17060945

18. Croce CM. Causes and consequences of microRNA dysregulation in cancer. Nat Rev Genet 2009; 10: 704–714. doi: 10.1038/nrg2634 PMID: 19763153

19. Winter J, Jung S, Keller S, Gregory RI, Diederichs S. Many roads to maturity: microRNA biogenesis pathways and their regulation. Nat Cell Biol 2009; 11: 228–234. doi: 10.1038/ncb0309-228 PMID: 19255566

20. Yang JS, Lai EC. Alternative miRNA biogenesis pathways and the interpretation of core miRNA pathway mutants. Mol Cell 2011; 43: 892–903. doi: 10.1016/j.molcel.2011.07.024 PMID: 21925378

21. Creighton CJ, Reid JG, Gunaratne PH. Expression profiling of microRNAs by deep sequencing. Brief Bioinform 2009; 10: 490–497. doi: 10.1093/bib/bbp019 PMID: 19332473

22. Griffiths-Jones S, Hui JH, Marco A, Ronshaugen M. MicroRNA evolution by arm switching. EMBO Rep 2011; 12: 172–177. doi: 10.1038/embor.2010.191 PMID: 21212805

23. Krol J, Loedige I, Filipowicz W. The widespread regulation of microRNA biogenesis, function and decay. Nat Rev Genet 2010; 11: 597–610. doi: 10.1038/nrg2843 PMID: 20661255

24. Khvorova A, Reynolds A, Jayasena SD. Functional siRNAs and miRNAs exhibit strand bias. Cell 2003; 115: 209–216. PMID: 14567918

25. Schwarz DS, Hutvagner G, Du T, Xu Z, Aronin N, et al. Asymmetry in the assembly of the RNAi enzyme complex. Cell 2003; 115: 199–208. PMID: 14567917

26. Chang HT, Li SC, Ho MR, Pan HW, Ger LP, et al. Comprehensive analysis of microRNAs in breast cancer. BMC Genomics 2012; 13 Suppl 7: S18. doi: 10.1186/1471-2164-13-S7-S18 PMID: 23281739

27. Chiang HR, Schoenfeld LW, Ruby JG, Auyeung VC, Spies N, et al. Mammalian microRNAs: experimental evaluation of novel and previously annotated genes. Genes Dev 2010; 24: 992–1009. doi: 10.1101/gad.1884710 PMID: 20413612

28. Cloonan N, Wani S, Xu Q, Gu J, Lea K, et al. MicroRNAs and their isomiRs function cooperatively to target common biological pathways. Genome Biol 2011; 12: R126. doi: 10.1186/gb-2011-12-12-r126 PMID: 22208850

29. Jagadeeswaran G, Zheng Y, Sumathipala N, Jiang H, Arrese EL, et al. Deep sequencing of small RNA libraries reveals dynamic regulation of conserved and novel microRNAs and microRNA-stars during silkworm development. BMC Genomics 2010; 11: 52. doi: 10.1186/1471-2164-11-52 PMID: 20089182

30. Li SC, Liao YL, Ho MR, Tsai KW, Lai CH, et al. miRNA arm selection and isomiR distribution in gastric cancer. BMC Genomics 2012; 13 Suppl 1: S13. doi: 10.1186/1471-2164-13-S1-S13 PMID: 22369582

31. Marco A, Hui JH, Ronshaugen M, Griffiths-Jones S. Functional shifts in insect microRNA evolution. Genome Biol Evol 2010; 2: 686–696. doi: 10.1093/gbe/evq053 PMID: 20817720

32. Ameres SL, Zamore PD. Diversifying microRNA sequence and function. Nat Rev Mol Cell Biol 2013; 14: 475–488. doi: 10.1038/nrm3611 PMID: 23800994

33. Glazov EA, Cottee PA, Barris WC, Moore RJ, Dalrymple BP, et al. A microRNA catalog of the developing chicken embryo identified by a deep sequencing approach. Genome Res 2008; 18: 957–964. doi: 10.1101/gr.074740.107 PMID: 18469162

34. Morin RD, O'Connor MD, Griffith M, Kuchenbauer F, Delaney A, et al. Application of massively parallel sequencing to microRNA profiling and discovery in human embryonic stem cells. Genome Res 2008; 18: 610–621. doi: 10.1101/gr.7179508 PMID: 18285502

35. Fernandez-Valverde SL, Taft RJ, Mattick JS. Dynamic isomiR regulation in Drosophila development. RNA 2010; 16: 1881–1888. doi: 10.1261/rna.2379610 PMID: 20805289

36. Lee LW, Zhang S, Etheridge A, Ma L, Martin D, et al. Complexity of the microRNA repertoire revealed by next-generation sequencing. RNA 2010; 16: 2170–2180. doi: 10.1261/rna.2225110 PMID: 20876832

37. Humphreys DT, Hynes CJ, Patel HR, Wei GH, Cannon L, et al. Complexity of murine cardiomyocyte miRNA biogenesis, sequence variant expression and function. PLoS One 2012; 7: e30933. doi: 10.1371/journal.pone.0030933 PMID: 22319597

38. Llorens F, Hummel M, Pantano L, Pastor X, Vivancos A, et al. Microarray and deep sequencing cross-platform analysis of the mirRNome and isomiR variation in response to epidermal growth factor. BMC Genomics 2013; 14: 371. doi: 10.1186/1471-2164-14-371 PMID: 23724959

39. Marti E, Pantano L, Banez-Coronel M, Llorens F, Minones-Moyano E, et al. A myriad of miRNA variants in control and Huntington's disease brain regions detected by massively parallel sequencing. Nucleic Acids Res 2010; 38: 7219–7235. doi: 10.1093/nar/gkq575 PMID: 20591823

40. Vaz C, Ahmad HM, Bharti R, Pandey P, Kumar L, et al. Analysis of the microRNA transcriptome and expression of different isomiRs in human peripheral blood mononuclear cells. BMC Res Notes 2013; 6: 390. doi: 10.1186/1756-0500-6-390 PMID: 24073671

41. Zhou H, Arcila ML, Li Z, Lee EJ, Henzler C, et al. Deep annotation of mouse iso-miR and iso-moR variation. Nucleic Acids Res 2012; 40: 5864–5875. doi: 10.1093/nar/gks247 PMID: 22434881

42. WHO (2013) Global Tuberculosis Report 2013. World Health Organization, Geneva.

43. Fu Y, Yi Z, Wu X, Li J, Xu F. Circulating microRNAs in patients with active pulmonary tuberculosis. J Clin Microbiol 2011; 49: 4246–4251. doi: 10.1128/JCM.05459-11 PMID: 21998423

44. Kumar R, Halder P, Sahu SK, Kumar M, Kumari M, et al. Identification of a novel role of ESAT-6-dependent miR-155 induction during infection of macrophages with Mycobacterium tuberculosis. Cell Microbiol 2012; 14: 1620–1631. doi: 10.1111/j.1462-5822.2012.01827.x PMID: 22712528

45. Liu Y, Wang X, Jiang J, Cao Z, Yang B, et al. Modulation of T cell cytokine production by miR-144* with elevated expression in patients with pulmonary tuberculosis. Mol Immunol 2011; 48: 1084–1090. doi: 10.1016/j.molimm.2011.02.001 PMID: 21367459

46. Ma F, Xu S, Liu X, Zhang Q, Xu X, et al. The microRNA miR-29 controls innate and adaptive immune responses to intracellular bacterial infection by targeting interferon-gamma. Nat Immunol 2011; 12: 861–869. doi: 10.1038/ni.2073 PMID: 21785411

47. Maertzdorf J, Weiner J 3rd, Mollenkopf HJ, Bauer T, Prasse A, et al. Common patterns and disease-related signatures in tuberculosis and sarcoidosis. Proc Natl Acad Sci U S A 2012; 109: 7853–7858. doi: 10.1073/pnas.1121072109 PMID: 22547807

48. Rajaram MV, Ni B, Morris JD, Brooks MN, Carlson TK, et al. Mycobacterium tuberculosis lipomannan blocks TNF biosynthesis by regulating macrophage MAPK-activated protein kinase 2 (MK2) and microRNA miR-125b. Proc Natl Acad Sci U S A 2011; 108: 17408–17413. doi: 10.1073/pnas.1112660108 PMID: 21969554

49. Sharbati J, Lewin A, Kutz-Lohroff B, Kamal E, Einspanier R, et al. Integrated microRNA-mRNA-analysis of human monocyte derived macrophages upon Mycobacterium avium subsp. hominissuis infection. PLoS One 2011; 6: e20258. doi: 10.1371/journal.pone.0020258 PMID: 21629653

50. Singh Y, Kaul V, Mehra A, Chatterjee S, Tousif S, et al. Mycobacterium tuberculosis controls microRNA-99b (miR-99b) expression in infected murine dendritic cells to modulate host immunity. J Biol Chem 2013; 288: 5056–5061. doi: 10.1074/jbc.C112.439778 PMID: 23233675

51. Spinelli SV, Diaz A, D'Attilio L, Marchesini MM, Bogue C, et al. Altered microRNA expression levels in mononuclear cells of patients with pulmonary and pleural tuberculosis and their relation with components of the immune response. Mol Immunol 2013; 53: 265–269. doi: 10.1016/j.molimm.2012.08.008 PMID: 22964481

52. Wang C, Yang S, Sun G, Tang X, Lu S, et al. Comparative miRNA expression profiles in individuals with latent and active tuberculosis. PLoS One 2011; 6: e25832. doi: 10.1371/journal.pone.0025832 PMID: 22003408

53. Wu J, Lu C, Diao N, Zhang S, Wang S, et al. Analysis of microRNA expression profiling identifies miR-155 and miR-155* as potential diagnostic markers for active tuberculosis: a preliminary study. Hum Immunol 2012; 73: 31–37. doi: 10.1016/j.humimm.2011.10.003 PMID: 22037148

54. Yi Z, Fu Y, Ji R, Li R, Guan Z. Altered microRNA signatures in sputum of patients with active pulmonary tuberculosis. PLoS One 2012; 7: e43184. doi: 10.1371/journal.pone.0043184 PMID: 22900099

55. Siddle KJ, Deschamps M, Tailleux L, Nedelec Y, Pothlichet J, et al. A genomic portrait of the genetic architecture and regulatory impact of microRNA expression in response to infection. Genome Res 2014; 24: 850–859. doi: 10.1101/gr.161471.113 PMID: 24482540

56. Friedlander MR, Mackowiak SD, Li N, Chen W, Rajewsky N. miRDeep2 accurately identifies known and hundreds of novel microRNA genes in seven animal clades. Nucleic Acids Res 2012; 40: 37–52. doi: 10.1093/nar/gkr688 PMID: 21911355

57. Anders S, Huber W. Differential expression analysis for sequence count data. Genome Biol 2010; 11: R106. doi: 10.1186/gb-2010-11-10-r106 PMID: 20979621

58. Ernst J, Bar-Joseph Z. STEM: a tool for the analysis of short time series gene expression data. BMC Bioinformatics 2006; 7: 191. PMID: 16597342

59. Ernst J, Nau GJ, Bar-Joseph Z. Clustering short time series gene expression data. Bioinformatics 2005; 21 Suppl 1: i159–168. PMID: 15961453
60. Yang JH, Li JH, Shao P, Zhou H, Chen YQ, et al. starBase: a database for exploring microRNA-mRNA interaction maps from Argonaute CLIP-Seq and Degradome-Seq data. Nucleic Acids Res 2011; 39: D202–209. doi: 10.1093/nar/gkq1056 PMID: 21037263
61. Langfelder P, Zhang B, Horvath S. Defining clusters from a hierarchical cluster tree: the Dynamic Tree Cut package for R. Bioinformatics 2008; 24: 719–720. PMID: 18024473
62. Yang JH, Li JH, Jiang S, Zhou H, Qu LH. ChIPBase: a database for decoding the transcriptional regulation of long non-coding RNA and microRNA genes from ChIP-Seq data. Nucleic Acids Res 2013; 41: D177–187. doi: 10.1093/nar/gks1060 PMID: 23161675
63. Barreiro LB, Tailleux L, Pai AA, Gicquel B, Marioni JC, et al. Deciphering the genetic architecture of variation in the immune response to Mycobacterium tuberculosis infection. Proc Natl Acad Sci U S A 2012; 109: 1204–1209. doi: 10.1073/pnas.1115761109 PMID: 22233810
64. Viboud GI, Bliska JB. Yersinia outer proteins: role in modulation of host cell signaling responses and pathogenesis. Annu Rev Microbiol 2005; 59: 69–89. PMID: 15847602
65. Nahid MA, Yao B, Dominguez-Gutierrez PR, Kesavalu L, Satoh M, et al. Regulation of TLR2-mediated tolerance and cross-tolerance through IRAK4 modulation by miR-132 and miR-212. J Immunol 2013; 190: 1250–1263. doi: 10.4049/jimmunol.1103060 PMID: 23264652
66. Pym AS, Brodin P, Brosch R, Huerre M, Cole ST. Loss of RD1 contributed to the attenuation of the live tuberculosis vaccines Mycobacterium bovis BCG and Mycobacterium microti. Mol Microbiol 2002; 46: 709–717. PMID: 12410828
67. Lewis BP, Burge CB, Bartel DP. Conserved seed pairing, often flanked by adenosines, indicates that thousands of human genes are microRNA targets. Cell 2005; 120: 15–20. PMID: 15652477
68. Bueno MJ, Perez de Castro I, Malumbres M. Control of cell proliferation pathways by microRNAs. Cell Cycle 2008; 7: 3143–3148. PMID: 18843198
69. Galindo CL, Rosenzweig JA, Kirtley ML, Chopra AK. Pathogenesis of Y. enterocolitica and Y. pseudotuberculosis in Human Yersiniosis. J Pathog 2011; 2011: 182051. doi: 10.4061/2011/182051 PMID: 22567322
70. van Kooyk Y, Geijtenbeek TB. DC-SIGN: escape mechanism for pathogens. Nat Rev Immunol 2003; 3: 697–709. PMID: 12949494
71. Jenner RG, Young RA. Insights into host responses against pathogens from transcriptional profiling. Nat Rev Microbiol 2005; 3: 281–294. PMID: 15806094
72. Amit I, Garber M, Chevrier N, Leite AP, Donner Y, et al. Unbiased reconstruction of a mammalian transcriptional network mediating pathogen responses. Science 2009; 326: 257–263. doi: 10.1126/science.1179050 PMID: 19729616
73. He X, Jing Z, Cheng G. MicroRNAs: new regulators of Toll-like receptor signalling pathways. Biomed Res Int 2014; 2014: 945169. doi: 10.1155/2014/945169 PMID: 24772440
74. O'Neill LA, Sheedy FJ, McCoy CE. MicroRNAs: the fine-tuners of Toll-like receptor signalling. Nat Rev Immunol 2011; 11: 163–175. doi: 10.1038/nri2957 PMID: 21331081
75. Chen CZ, Schaffert S, Fragoso R, Loh C. Regulation of immune responses and tolerance: the microRNA perspective. Immunol Rev 2013; 253: 112–128. doi: 10.1111/imr.12060 PMID: 23550642
76. Li Y, Shi X. MicroRNAs in the regulation of TLR and RIG-I pathways. Cell Mol Immunol 2013; 10: 65–71. doi: 10.1038/cmi.2012.55 PMID: 23262976
77. Alexiou P, Maragkakis M, Papadopoulos GL, Reczko M, Hatzigeorgiou AG. Lost in translation: an assessment and perspective for computational microRNA target identification. Bioinformatics 2009; 25: 3049–3055. doi: 10.1093/bioinformatics/btp565 PMID: 19789267
78. Martinez-Sanchez A, Murphy CL. MicroRNA Target Identification-Experimental Approaches. Biology (Basel) 2013; 2: 189–205. doi: 10.3390/biology2010189 PMID: 24832658
79. Frigui W, Bottai D, Majlessi L, Monot M, Josselin E, et al. Control of M. tuberculosis ESAT-6 secretion and specific T cell recognition by PhoP. PLoS Pathog 2008; 4: e33. doi: 10.1371/journal.ppat.0040033 PMID: 18282096
80. Taganov KD, Boldin MP, Chang KJ, Baltimore D. NF-kappaB-dependent induction of microRNA miR-146, an inhibitor targeted to signaling proteins of innate immune responses. Proc Natl Acad Sci U S A 2006; 103: 12481–12486. PMID: 16885212
81. Portevin D, Gagneux S, Comas I, Young D. Human macrophage responses to clinical isolates from the Mycobacterium tuberculosis complex discriminate between ancient and modern lineages. PLoS Pathog 2011; 7: e1001307. doi: 10.1371/journal.ppat.1001307 PMID: 21408618

82. Lewis BP, Shih IH, Jones-Rhoades MW, Bartel DP, Burge CB. Prediction of mammalian microRNA targets. Cell 2003; 115: 787–798. PMID: 14697198

83. Burroughs AM, Ando Y, de Hoon MJ, Tomaru Y, Nishibu T, et al. A comprehensive survey of 3' animal miRNA modification events and a possible role for 3' adenylation in modulating miRNA targeting effectiveness. Genome Res 2010; 20: 1398–1410. doi: 10.1101/gr.106054.110 PMID: 20719920

84. Neilsen CT, Goodall GJ, Bracken CP. IsomiRs—the overlooked repertoire in the dynamic microRNAome. Trends Genet 2012; 28: 544–549. doi: 10.1016/j.tig.2012.07.005 PMID: 22883467

85. Jones MR, Quinton LJ, Blahna MT, Neilson JR, Fu S, et al. Zcchc11-dependent uridylation of microRNA directs cytokine expression. Nat Cell Biol 2009; 11: 1157–1163. doi: 10.1038/ncb1931 PMID: 19701194

86. Katoh T, Sakaguchi Y, Miyauchi K, Suzuki T, Kashiwabara S, et al. Selective stabilization of mammalian microRNAs by 3' adenylation mediated by the cytoplasmic poly(A) polymerase GLD-2. Genes Dev 2009; 23: 433–438. doi: 10.1101/gad.1761509 PMID: 19240131

87. Backes S, Shapiro JS, Sabin LR, Pham AM, Reyes I, et al. Degradation of host microRNAs by poxvirus poly(A) polymerase reveals terminal RNA methylation as a protective antiviral mechanism. Cell Host Microbe 2012; 12: 200–210. doi: 10.1016/j.chom.2012.05.019 PMID: 22901540

88. Bartel DP. MicroRNAs: target recognition and regulatory functions. Cell 2009; 136: 215–233. doi: 10.1016/j.cell.2009.01.002 PMID: 19167326

89. Morgan M, Anders S, Lawrence M, Aboyoun P, Pages H, et al. ShortRead: a bioconductor package for input, quality assessment and exploration of high-throughput sequence data. Bioinformatics 2009; 25: 2607–2608. doi: 10.1093/bioinformatics/btp450 PMID: 19654119

90. Langmead B, Trapnell C, Pop M, Salzberg SL. Ultrafast and memory-efficient alignment of short DNA sequences to the human genome. Genome Biol 2009; 10: R25. doi: 10.1186/gb-2009-10-3-r25 PMID: 19261174

91. de Hoon MJ, Taft RJ, Hashimoto T, Kanamori-Katayama M, Kawaji H, et al. Cross-mapping and the identification of editing sites in mature microRNAs in high-throughput sequencing libraries. Genome Res 2010; 20: 257–264. doi: 10.1101/gr.095273.109 PMID: 20051556

92. Quinlan AR, Hall IM. BEDTools: a flexible suite of utilities for comparing genomic features. Bioinformatics 2010; 26: 841–842. doi: 10.1093/bioinformatics/btq033 PMID: 20110278

93. Fournier D, Skaug H, Ancheta J, Ianelli J, Magnusson A, et al. AD Model Builder: using automatic differentiation for statistical inference of highly parameterized complex nonlinear models. Optimization Methods and Software 2012; 27: 233–249.

94. Abecasis GR, Auton A, Brooks LD, DePristo MA, Durbin RM, et al. An integrated map of genetic variation from 1,092 human genomes. Nature 2012; 491: 56–65. doi: 10.1038/nature11632 PMID: 23128226

95. Excoffier L, Smouse PE, Quattro JM. Analysis of molecular variance inferred from metric distances among DNA haplotypes: application to human mitochondrial DNA restriction data. Genetics 1992; 131: 479–491. PMID: 1644282

96. Weir CL, Cockerham CC. Estimating F-statistics for the analysis of population structure. Evolution 1984; 38: 1358–1370.

97. Redon R, Ishikawa S, Fitch KR, Feuk L, Perry GH, et al. Global variation in copy number in the human genome. Nature 2006; 444: 444–454. PMID: 17122850

98. Grimson A, Farh KK, Johnston WK, Garrett-Engele P, Lim LP, et al. MicroRNA targeting specificity in mammals: determinants beyond seed pairing. Mol Cell 2007; 27: 91–105. PMID: 17612493

99. Langfelder P, Horvath S. WGCNA: an R package for weighted correlation network analysis. BMC Bioinformatics 2008; 9: 559. doi: 10.1186/1471-2105-9-559 PMID: 19114008

100. Langfelder P, Horvath S. Fast R Functions for Robust Correlations and Hierarchical Clustering. J Stat Softw 2012; 46. PMID: 23050260

101. Livak KJ, Schmittgen TD. Analysis of relative gene expression data using real-time quantitative PCR and the 2(-Delta Delta C(T)) Method. Methods 2001; 25: 402–408. PMID: 11846609
